# Genome-wide identification of the *SWEET* gene family mediating the cold stress response in *Prunus mume*

**DOI:** 10.7717/peerj.13273

**Published:** 2022-05-03

**Authors:** Zhenying Wen, Mingyu Li, Juan Meng, Ping Li, Tangren Cheng, Qixiang Zhang, Lidan Sun

**Affiliations:** Beijing Key Laboratory of Ornamental Plants Germplasm Innovation & Molecular Breeding, School of Landscape Architecture, National Engineering Research Center for Floriculture, Beijing Laboratory of Urban and Rural Ecological Environment, Beijing Forestry University, Beijing, China

**Keywords:** *Prunus mume*, *SWEET* gene family, Gene expression, Cold response

## Abstract

The Sugars Will Eventually be Exported Transporter (SWEET) gene family encodes a family of sugar transporters that play essential roles in plant growth, reproduction, and biotic and abiotic stresses. *Prunus mume* is a considerable ornamental wood plant with high edible and medicinal values; however, its lack of tolerance to low temperature has severely limited its geographical distribution. To investigate whether this gene family mediates the response of *P. mume* to cold stress, we identified that the *P. mume* gene family consists of 17 members and divided the family members into four groups. Sixteen of these genes were anchored on six chromosomes, and one gene was anchored on the scaffold with four pairs of segmental gene duplications and two pairs of tandem gene duplications. *Cis*-acting regulatory element analysis indicated that the *PmSWEET* genes are potentially involved in *P. mume* development, including potentially regulating roles in procedure, such as circadian control, abscisic acid-response and light-response, and responses to numerous stresses, such as low-temperature and drought. We performed low-temperature treatment in the cold-tolerant cultivar ‘Songchun’ and cold-sensitive cultivar ‘Zaolve’ and found that the expression of four of 17 *PmSWEETs* was either upregulated or downregulated with prolonged treatment times. This finding indicates that these family members may potentially play a role in cold stress responses in *P. mume*. Our study provides a basis for further investigation of the role of SWEET proteins in the development of *P. mume* and its responses to cold stress.

## Introduction

Sucrose is the main carbohydrate in most plants; it is synthesized in the leaves during photosynthesis and then transported by phloem sap to storage organs, such as roots, stems, flowers, seeds and fruits (Rennie & Turgeon, 2009; Lemoine et al., 2013). Sucrose provides energy and carbon sources for plants and acts as an important signal and resistance molecule that participates in the normal growth of higher plants (Chen et al., 2015). However, sucrose must be assisted by appropriate sugar transporters as it cannot be transported independently to the storage organs (Ainsworth & Bush, 2011). At present, three transporter families have been identified as essential sugar transporters: monosaccharide transporters (MSTs), sucrose transporters (SUTs), and Sugar Will Eventually be Exported Transporters (SWEETs) (Chen et al., 2010, 2015; Eom et al., 2015). Of these three families, *SWEETs* were the final gene family to be uncovered and were first identified by Chen et al. (2010) in *Arabidopsis*. SWEET proteins act as sugar transporters that mediate the inflow or outflow of phloem parenchyma sugar into the phloem apoplast (Slewinski, 2011; Braun, 2012; Chen, 2014). Unlike the SUT and MST families, which require energy to transport sugar across the plasma membrane (Maynard & Lucas, 1982; Lemoine, 2000), SWEET proteins promote the diffusion of sugar across concentration gradients at the cellular membrane or vacuolar membrane, regardless of the proton gradient or pH of the cellular environment (Chen et al., 2012, 2015).

SWEET proteins are characterized by conserved MtN3_saliva (MtN3_slv) transmembrane (TM) domains (Chen et al., 2012), also known as PQ-loop repeats (Eom et al., 2015; Feng & Frommer, 2015). SWEETs in eukaryotes commonly consist of seven transmembrane helices (TMHs), which contain a pair of 3-TMH repeats detached by an added helix (Xuan et al., 2013), and this structure has been described as the “3-1-3” TM SWEET structure (Chen et al., 2010). In contrast to the structure of eukaryote SWEET proteins, prokaryote SWEET proteins, known as SemiSWEETs, are composed of only three TMHs (Xuan et al., 2013). In eukaryotes, proteins that contain 6 or 7 TMHs are prevalent, but SemiSWEETs with 3 or 4 TMHs have also been detected in plant genomes. In a study of *SWEET* genes from 25 plant genomes, 140 of the 411 SWEET sugar transporters identified were classed as being a semiSWEET; with all of the identified semiSWEETs either lacking the first or second 3-TM domain or which only existed in a partial form (Patil et al., 2015). This data therefore demonstrates that the presence of semiSWEETs in higher plant genomes is not unusual, and further, that SWEETs may in actual fact have formed by direct fusion from SemiSWEETs (Jia et al., 2017). In addition, a novel extraSWEET protein consisting of 14 and 15 TMHs has been reported from *Vitis vinifera* (Patil et al., 2015) and *Oryza punctata* (Jia et al., 2017); it is speculated that this extraSWEET may have formed from the duplication of a 7 TMH *SWEET* gene in these two species. Recent research on 3,249 SWEET proteins also identified a superSWEET with >18 TMHs in oomycetes, which carry 5–8 repeats of a semiSWEET (Jia et al., 2017). According to phylogenetic analysis, the *SWEET* genes in *Arabidopsis* can be divided into four clades: Clade I (*SWEET1*–*3*) and Clade II (*SWEET4*–*8*) mainly transport glucose, while Clade I *SWEETs* also have the ability to transport hexose (Chen et al., 2010; Lin et al., 2014). Clade III members (*SWEET9*–*15*) mainly transport sucrose (Chen et al., 2012; Eom et al., 2015), and Clade IV members (*SWEET16*–*17*), which are located on the tonoplast membrane, mainly transport fructose (Eom et al., 2015). The phylogenetic relationships of the *SWEET* genes described hereafter are all based on results from *Arabidopsis*.

Advances in whole-genome sequencing have enabled genome-wide identification of *SWEET* genes in numerous species. These include important crops, fruits and vegetables, such as rice (*Oryza sativa*) (Yuan & Wang, 2013), sorghum (*Sorghum bicolor*) (Mizuno, Kasuga & Kawahigashi, 2016), soybean (*Glycine max*) (Patil et al., 2015), apple (*Malus domestica*) (Wei et al., 2014), grape (*Vitis vinifera*) (Chong et al., 2014), banana (*Musa acuminate*) (Miao et al., 2017), tomato (*Solanum lycopersicum*) (Feng et al., 2015), rapeseed (*Brassica napus*) (Jian et al., 2016), potato (*Solanum tuberosum*) (Li et al., 2020) and valencia sweet orange (*Citrus sinensis*) (Yao et al., 2021). Additionally, many *SWEET* genes have been confirmed to play diverse and complex roles in physiological processes, such as nectar secretion (Ge et al., 2000; Lin et al., 2014), pollen development (Sun et al., 2013), senescence (Quirino, Normanly & Amasino, 1999), and seed filling (Sosso et al., 2015). Moreover, *SWEET* genes are also involved in biotic and abiotic stress responses (Yuan & Wang, 2013), including the reaction of plants to stress at low temperatures. For example, overexpression of *AtSWEET16* and *AtSWEET17* increases cold tolerance (Chardon et al., 2013; Klemens et al., 2013; Guo et al., 2014); overexpression of *AtSWEET4* increases plant biomass and its resistance to frost (Chong et al., 2014; Liu et al., 2016); and *AtSWEET11* and *AtSWEET12* are involved in responses to cold or dehydration stress (Le Hir et al., 2015; Durand et al., 2016). *AtSWEET15* is also known as *SAG29* (where SAG stands for senescence-associated gene); however, its transcription level gradually increases at low temperature, high salinity, and drought during natural leaf senescence (Quirino, Normanly & Amasino, 1999). Cold stress significantly inhibits the expression of *CsSWEET2*, *CsSWEET3*, and *CsSWEET16* in *Camellia sinensis* (tea plant), while the expression of *CsSWEET1* and *CsSWEET17* increases sharply (Yue et al., 2015). A functional study of *CsSWEET16* in *C. sinensis* revealed that it is located in the vacuolar membrane, and furthermore, in transgenic *Arabidopsis* plants, *CsSWEET16* expression regulates cold resistance (Wang et al., 2018). The transcriptional activity of many *SlSWEET* genes increases under low-temperature stress in tomato (Feng et al., 2015). Studies have shown that expression of the *MaSWEET* gene in banana is upregulated in response to low temperature, salt, and osmotic stress (Miao et al., 2017). Using genome-wide analysis of the *BoSWEET* gene in *Brassica oleracea* var. *capitata* (wild cabbage), five possible candidate genes were found to promote sugar transport and thereby enhance chilling tolerance of wild cabbage (Zhang et al., 2019).

*Prunus mume* is a traditional flower native to southwest China and the middle and lower reaches of the Yangtze River. In northern China, low temperatures severely limit the growth and distribution of this species. Although SWEET sugar transporters have been associated with responses to cold stress in other species, little is known about the role of *PmSWEET*s in cold responses in *P. mume*. This study aims to conduct a genome-wide analysis of the *SWEET* gene family in *P. mume*, with a specific focus on *SWEET* gene transcriptional responses to cold stress, providing a starting point to perform a detailed study of the potential functional roles of *SWEET* gene family members in *P. mume*.

## Materials and Methods

### Plant genomic resources

To explore the phylogeny of the *SWEET* genes in *P. mume* and other species, we downloaded SWEET proteins from two model plants (*Arabidopsis thaliana* and *Oryza sativa*, representing dicotyledons and monocotyledons, respectively) and eight other Rosaceae species. The protein sequences of 17 *At*SWEETs and 21 *Os*SWEETs were downloaded from the TAIR 10 database (http://www.arabidopsis.org/) and TIGR (http://rice.plantbiology.msu.edu/), respectively. The *P. mume* genome sequence and annotation files were obtained from the *P. mume* genome project (https://github.com/lileiting/prunusmumegenome); the genomes of eight other Rosaceae species, including *Malus domestica* (Daccord et al., 2017), *Prunus avium* (Shirasawa et al., 2017), *Prunus persica* (Verde et al., 2013), *Prunus yedoensis* (Baek et al., 2018), *Pyrus communis* (Linsmith et al., 2019), *Rosa chinensis* (Raymond et al., 2018), *Prunus salicina* (Liu et al., 2020), and *Prunus armeniaca* (Jiang et al., 2019), were downloaded from the Genome Database for Rosaceae (https://www.rosaceae.org/).

### Identification of *SWEET* genes in *P. mume* and other species

The hidden Markov model (HMM) profiles of the MtN3_slv domain for the *SWEET* gene family (PF03083) were downloaded from the Pfam database (http://pfam.xfam.org/) and used as queries to search for SWEET proteins in the proteomes of *P. mume* and other species with HMMER software (version 3.1b2, http://hmmer.org/) (Finn et al., 2015). To ensure confidence, the E-value cutoff was set at 10^−5^. Then, all putative SWEET proteins were screened to confirm the presence of the MtN3_slv domain by SMART (http://smart.embl-heidelberg.de/), the Pfam database (http://pfam.xfam.org/) and NCBI-CDD (https://www.ncbi.nlm.nih.gov/cdd), and sequences with MtN3_slv domain were retained.

The *SWEET* genes were named based on their location information in the *P. mume* genome. In addition, the number of amino acids, molecular weight (MW) and isoelectric point (pI) were calculated using the online ExPASy program (https://www.expasy.org/). The distributions of TM helices were predicted by use of the TMHMM Server v. 2.0 (https://services.healthtech.dtu.dk/service.php?TMHMM-2.0).

### Phylogenetic and conserved domain analysis

To examine the phylogeny between *SWEET* genes in *P. mume* and other species, alignment of full-length SWEET protein sequences from three species (*P. mume*, *A. thaliana*, and *O. sativa*) and eight Rosaceae species was performed by using MAFFT software with the FFT-NS-1 strategy (Katoh & Standley, 2013). Subsequently, maximum likelihood (ML) phylogenetic trees were constructed using FastTree (version 2.1.11) (Price, Dehal & Arkin, 2010) with default parameters. Then, iTOL v4.0 (https://itol.embl.de/itol.cgi) (Letunic & Bork, 2019) and AI CS6 software were used to annotate and embellish the phylogenetic tree.

### Conserved motif and gene structure analysis

The conserved motifs of each identified *Pm*SWEET protein was predicted by MEME Suite Version 5.3.3 (https://meme-suite.org/meme/tools/meme) (Bailey et al., 2009), where the maximum number of motifs for the conserved domains was set to 10, motif width was set to 6–50 amino acids, and the residuals were designated as the default parameters. Gene structure data was extracted from the *P. mume* genome gff file, visualized using TBtools software (Chen et al., 2020), and then edited in AI CS6 software.

### Chromosome location, duplication and synteny analysis

The location and chromosome length information of each *PmSWEET* gene was obtained from the gff file downloaded from the *P. mume* genome project (https://github.com/lileiting/prunusmumegenome). A chromosomal location figure was drawn using the online tool MG2C (http://mg2c.iask.in/mg2c_v2.0/). Gene tandem and segment replication events were analyzed using the Multiple Collinearity Scan Toolkit (MCScanX) and Circos in TBtools, respectively, with the default parameters. The synteny of the *PmSWEETs* across *A. thaliana*, *P. armeniaca*, and *P. salicina* was mapped using MCScanX in TBtools. The Ks and Ka values for duplicated gene pairs were calculated based on the coding sequence alignments using the Ka/Ks calculator in TBtools. According to two ordinary rates (λ) of 1.5 × 10^–8^ and 6.1 × 10^–9^ substitutions per site per year (Lynch & Conery, 2000; Blanc & Wolfe, 2004), the formula *t* = Ks/2λ × 10^–6^ Mya was used to calculate the divergence time.

### *Cis*-acting element analysis of *PmSWEET* gene promoter regions

The upstream genomic sequence (2.0 kb) of each identified *PmSWEET* gene was retrieved from the genomic sequence data in TBtools and then submitted to the PlantCARE database (http://bioinformatics.psb.ugent.be/webtools/plantcare/html/) (Lescot et al., 2002) for *cis*-acting element analysis. We finally selected 12 elements, including those induced by hormones, such as methyl jasmonate (MeJA)-responsive, abscisic acid (ABA)-responsive, and stress-responsive elements; the stress-responsive factors included those involved in defense and stress, low temperature, and light. By combining these data with phylogenetic tree information (nwk file), the map was constructed by TBtools and edited by AI CS6 software.

### *PmSWEET* genes expression analysis

To investigate the function of *PmSWEETs* involved in tissue development and cold tolerance, we used root, stem, leaf, flower bud and fruit data from RNA sequencing (Zhang et al., 2012) to analyze the *PmSWEET* expression patterns in different tissues and then used flower bud dormancy data from RNA sequencing of *P. mume* (‘Zaolve’) (Zhang et al., 2018) to analyze *PmSWEET* responses to low temperature from November to February. Furthermore, we explored the expression of *SWEET* gene family members in the stem of *P. mume* (‘Songchun’) in geographically distinct locations, including Beijing (BJ, N39°54′, E116°28′), Chifeng (CF, N42°17′, E118°58′) and Gongzhuling (GZL, N43°42′, E124°47′) and for three different periods of the year, including cold acclimation (October, autumn), the final period of endo-dormancy (January, winter), and deacclimation (March, spring) (Jiang, 2020). TBtools (Chen et al., 2020) was used to create the heatmap.

### qRT–PCR analysis of *PmSWEET* genes

To examine the response of *PmSWEET* to low temperature, the annual branches of the cold-sensitive cultivar ‘Zaolve’ and the cold-tolerant cultivar ‘Songchun’ were collected. Before chilling treatment, the shoots were incubated overnight at 22 °C and then transferred to 4 °C for 0, 1, 4, 6, 12, 24, 48, and 72 h under long-day conditions (16-h light/8-h dark). The stems were collected immediately and transferred to liquid nitrogen until their longterm storage at –80 °C in readiness for RNA extraction. Each treatment had three biological replicates.

Total RNA of each sample was extracted using the RNAprep Pure Plant Plus Kit (Tiangen, Beijing, China). Complementary cDNA was synthesized using ReverTra Ace^®^ qPCR RT Master Mix with gDNA Remover (Toyobo, Osaka, Japan). The specific primers were designed by Primer 3 (https://bioinfo.ut.ee/primer3-0.4.0/) based on the cDNA sequences (Table S1). The expression levels of *PmSWEETs* at low temperature were analyzed using quantitative real-time polymerase chain reaction (qRT–PCR) with a PikoReal real-time PCR system (Thermo Fisher Scientific, San Francisco, CA, USA) with SYBR^®^ Green Premix *Pro Taq* HS qPCR kit (Accurate Biology, Changsha, China). The reactions were performed in a 10 μL volume, including 5.0 μL SYBR^®^ Green Premix *Pro Taq* HS qPCR master mix, 0.5 μL each of forward and reverse primer, 1.0 μL of cDNA and 3.0 μL of ddH_2_O. The reactions were performed according to the following procedure: 95 °C for 30 s, followed by 40 cycles of 95 °C for 5 s and 60 °C for 30 s. Through the use of the *PHOSPHATASE 2A* (*PP2A*) gene of *P. mume* as the reference gene, the relative expression was calculated by using the delta-delta CT method (Livak & Schmittgen, 2001). Each qRT-PCR was conducted *via* the use of three biological replicates. The statistical analyses of ‘Zaolve’ and ‘Songchun’ were conducted independently using SPSS22.0, the one-way ANOVA analysis of variance was calculated by least significant difference (LSD) and Student–Newman–Keuls test with significant difference at level *p* = 0.05. GraphPad Prism6 software was used to draw the diagram.

## Results

### Identification of members of the *Prunus mume SWEET* gene family

A total of 17 nonredundant *PmSWEETs* were detected in the *P. mume* genome (sequence information is shown in File S1), and 175 *SWEETs* were detected in the eight other species of Rosaceae, including 16 *SWEET* genes in *P. armeniaca*, 19 in *P. avium*, 19 in *P. persica*, 19 in *P. salicina*, 16 in *P. yedoensis*, 21 in *P. communis*, 29 in *M. domestica*, and 36 in *R. chinensis* with rigorous filtering. All the newly identified *SWEET* genes were named according to their chromosome location (Table 1; Table S2). We determined that candidates with at least one MtN3_slv domain were “genuine” *SWEET* genes (domain architecture of *PmSWEETs* is shown in File S2). The number of amino acids, molecular weight (MW), and isoelectric point (pI) were calculated on the basis of the protein sequence of each identified SWEET. As exhibited in Table 1, the predicted *Pm*SWEET proteins ranged from 105 (*PmSWEET14*) to 580 (*PmSWEET8*) amino acids in length, with relative molecular weights ranging from 15.96 kDa (*PmSWEET11*) to 63.43 kDa (*PmSWEET8*), and theoretical pIs ranging from 8.30 (*PmSWEET4*) to 9.76 (*PmSWEET3*). The MW and pI of family member *PmSWEET14* could not be determined using this approach however due to the presence of four consecutive undefined amino acids (Table 1). Through prediction and analysis of TMHs of the 17 identified *PmSWEETs*, we found that these *Pm*SWEET proteins were predicted to have 2–7 TMHs. Surprisingly, only seven members of the *P. mume SWEET* gene family were determined to possess standard 7 TMHs, most other *SWEETs* have fewer than 7 TMHs. Detailed location information of the TMHs is shown in Table S3 and Fig. S1.

**Table 1:** The *PmSWEET* gene family members in *P. mume*.

Name	Gene ID	Clade	CDS (bp)	No. of amino acids	Molecular weight (kDa)	Theoretical pI	TMHs	No. of MtN3/saliva domain	Locus
*PmSWEET1*	Pm007067	III	849	282	31.38	8.34	7	2	Pa2:21184396…21186332
*PmSWEET2*	Pm008206	IV	759	252	27.74	8.50	7	2	Pa2:31718730…31721555
*PmSWEET3*	Pm010330	I	1248	415	46.25	9.76	7	2	Pa3:3891190…3895205
*PmSWEET4*	Pm011260	I	708	235	26.45	8.30	7	2	Pa3:9921623…9924001
*PmSWEET5*	Pm013198	II	519	172	19.42	8.97	5	1	Pa4:2433448…2434735
*PmSWEET6*	Pm015728	II	708	235	25.67	9.21	5	2	Pa4:21122646…21124537
*PmSWEET7*	Pm017566	IV	735	244	26.99	9.14	7	2	Pa5:12327097…12328384
*PmSWEET8*	Pm018875	III	1743	580	63.43	8.34	6	2	Pa5:20984940…20990591
*PmSWEET9*	Pm019954	III	828	275	30.68	9.20	7	2	Pa6:436315…437664
*PmSWEET10*	Pm021931	II	708	235	26.60	8.59	6	2	Pa6:12459796…12461199
*PmSWEET11*	Pm022695	I	417	138	15.96	9.74	3	1	Pa6:19934418…19935334
*PmSWEET12*	Pm022696	I	651	216	23.21	8.78	5	2	Pa6:19944525…19945680
*PmSWEET13*	Pm024167	II	780	259	28.66	9.37	6	2	Pa7:10796671…10798904
*PmSWEET14*	Pm024554	III	318	105	–	–	2	1	Pa7:13005181…13005663
*PmSWEET15*	Pm024555	III	891	296	33.14	8.61	7	2	Pa7:13012731…13014646
*PmSWEET16*	Pm024712	II	639	212	23.95	8.37	5	2	Pa7:13852243…13854234
*PmSWEET17*	Pm030352	I	510	169	19.26	9.14	4	1	scaffold54:138478…139392

### Phylogenetic analysis and classification of *SWEET* genes

**Figure 1:**
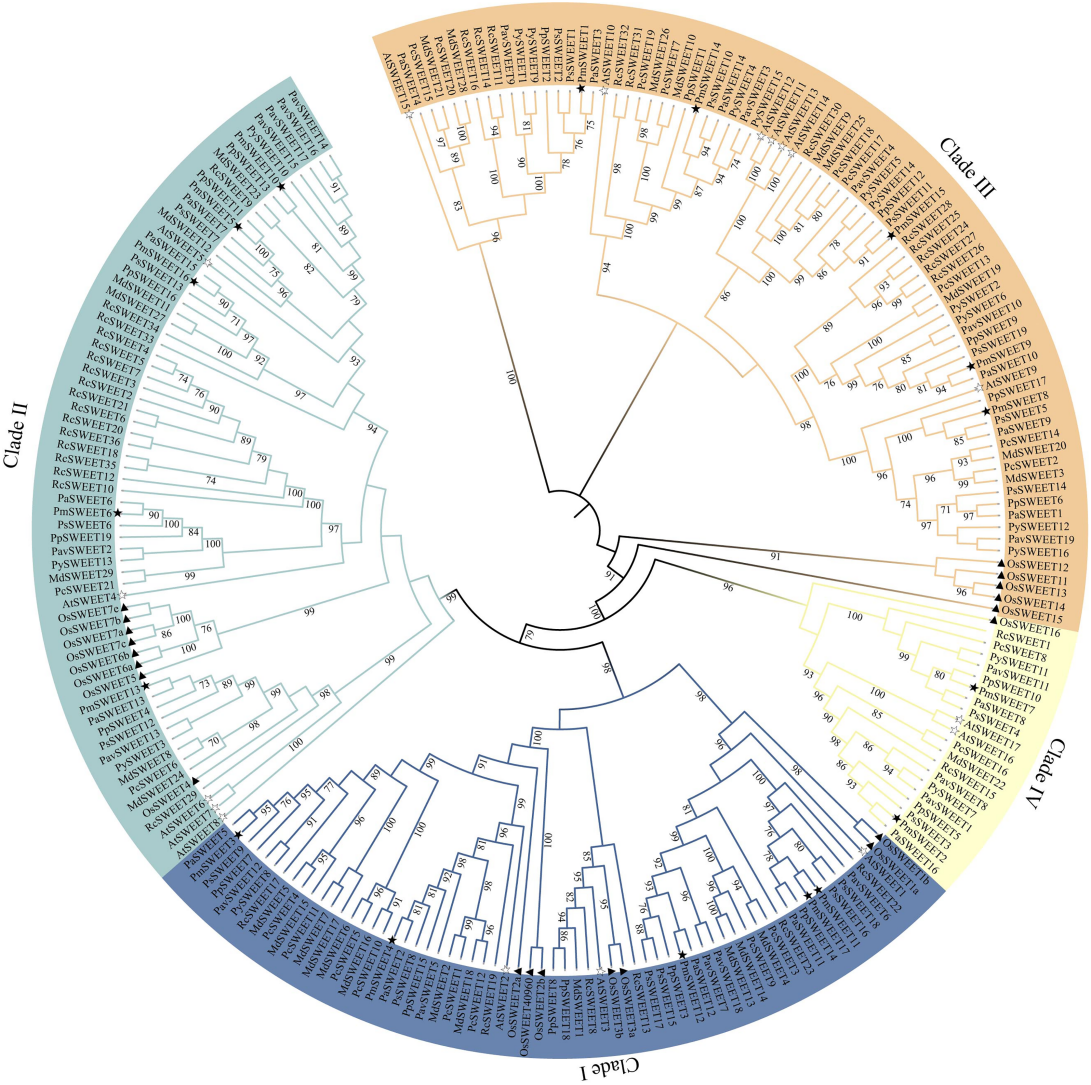
Phylogenetic tree of SWEET sequences from *P. mume* and other plant species. Clades I, II, III, and IV are indicated by blue, indigo, orange and pale yellow branch lines, respectively. At, *A. thaliana*; Os, *O. sativa*; Pa, *P. armeniaca*; Pav, *P. avium*; Pc, *P. communis*; Pm, *P. mume*; Pp, *P. persica*; Ps, *P. salicina*; Py, *P. yedoensis* var. *nudiflora*; Md, *M. domestica*; Rc, *R. chinensis*.

To better understand the evolution of homologous *SWEET* genes, we used the ML method to create a phylogenetic tree of all SWEET sequences from *A. thaliana* (model dicots), *O. sativa* (model monocots), and *P. mume*. According to previously reported *AtSWEETs* and *OsSWEETs* (Chen et al., 2010; Yuan & Wang, 2013), the 17 identified *PmSWEETs* were divided into four clades (*i.e*., Clade I, Clade II, Clade III, and Clade IV) (Fig. S2). To investigate the evolutionary relationships between *PmSWEETs* and the *SWEETs* of other species, a ML phylogenetic tree of *SWEETs* from 11 species, including 8 other Rosaceae species, was constructed. All members of the *SWEET* gene family in the 11 species were divided into four clades (Fig. 1). The largest clade was Clade III, which comprised five *OsSWEET* genes, seven *AtSWEET* genes, and 68 Rosaceae *SWEET* genes; the specific number of genes is shown in Table S4. The smallest clade was Clade IV, which consisted of only two *A. thaliana SWEET* genes, one *O. sativa SWEET* gene, and 18 Rosaceae *SWEET* genes (Table S4), a finding which shows that the *SWEET* genes are not evenly distributed across the four constructed clades. The numbers of genes in Clade I, II and III varied greatly, suggesting that the *SWEET* gene family expanded, especially in Clades I, II and III, during Rosaceae evolution. The *SWEETs* of Rosaceae were distributed uniformly across each small clade, whereas *SWEETs* from *O. sativa* tended to cluster together. The *PmSWEETs*, *PpSWEETs*, and *PavSWEETs* were clustered together and had similar distributions in the phylogenetic tree.

### Conserved motif and gene structure analysis

**Figure 2:**
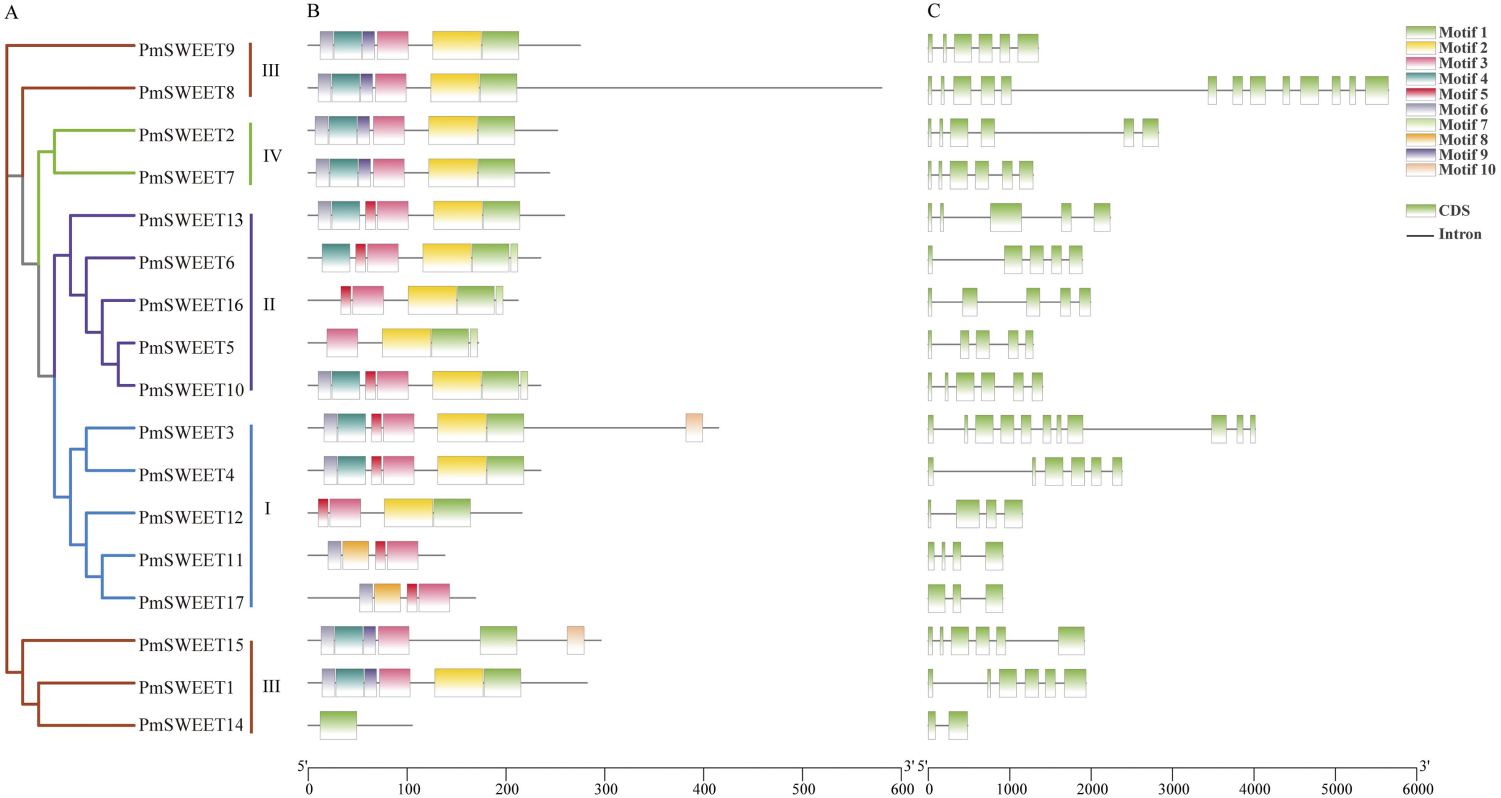
Phylogenetic relationship, conserved motif and gene structure analysis of *PmSWEET* genes. (A) The ML phylogenetic tree of *PmSWEET* genes. The *SWEET* genes were grouped into four clades, and blue, purple, red, and green represents Clades I, II, III, and IV, respectively. (B) The motif composition of *Pm*SWEET proteins. Ten motifs were displayed in different colored Rectangles. Motif1, GVVWFLYGLLKKDLFIAIPNGLGFJLGLVQLILYAIYR; Motif2, TKKRSLIVGIJCIVFNIIMYASPLTIMKLVIKTKSVEYMPFYLSLFLFLN; Motif3, LVITINGFGAVIELIYJAIFIIYAPKKKRKKI; Motif4, APVPTFYRIIKKKSTEEFQSVPYVAALLN; Motif5, WYGMPFVHPDN; Motif6, FGILGNIISFLLFL; Motif7, STNWDDDD; Motif8, PMTTLKRIMKKNEFTEQYLSGIPYLMT; Motif9, AMLWLYYGLLKPN; Motif10, NCZGCKDQYQHPQKCCKE. Detailed information is shown with logos obtained from the MEME Suite website in Fig. S3. (C) Exon-intron organization of *PmSWEET* genes. Green and black correspond to exons and introns, respectively.

To explore the sequence features of *Pm*SWEET proteins, MEME software and TBtools were used to predict and draw conserved domains. As a consequence, 10 distinct motifs were detected in SWEET proteins (Fig. 2B), and a schematic diagram of *Pm*SWEET protein motifs is shown in Fig. S3. The number of *Pm*SWEETs motifs was quite distinct, ranging from 1 to 7. Of them, 12 *Pm*SWEETs contained more than four motifs, 4 *Pm*SWEETs harbored four motifs, and *Pm*SWEET14 contained only one motif. Motifs 1, 2, 3, 4 and 6 were highly conserved and present in 15 *Pm*SWEET, 13 *Pm*SWEET, 16 *Pm*SWEET, 11 *Pm*SWEET and 12 *Pm*SWEET proteins, respectively; while motifs 7, 8 and 10 were relatively unique and existed in only 4 *Pm*SWEET, 2 *Pm*SWEET and 2 *Pm*SWEET proteins, respectively. Intriguingly, aside from some unusual proteins, SWEET members of the same clade had similar conserved motifs, suggesting that they might have similar functions.

To elucidate the structural characteristics of the *PmSWEETs*, the exon-intron structure was further analyzed. As shown in Fig. 2C, *PmSWEETs* in Clade II (except *PmSWEET10*) contained four introns. *PmSWEET1*, *PmSWEET9*, and *PmSWEET15* in Clade III had five introns, *PmSWEET8* contained the largest number of introns (12 introns), while *PmSWEET14* contained only one intron. All *PmSWEETs* in Clade IV had five introns. The number of introns in Clade I varied from just 2 to 10, *PmSWEET17* had two introns, *PmSWEET4* contained five introns, *PmSWEET11* and *PmSWEET12* contained three introns, *PmSWEET3* had 10 introns. These results indicated that aside from some unique gene family members, genes clustered together generally exhibited similar gene structures.

### Chromosomal distribution and tandem duplication of *PmSWEET* gene family members

**Figure 3:**
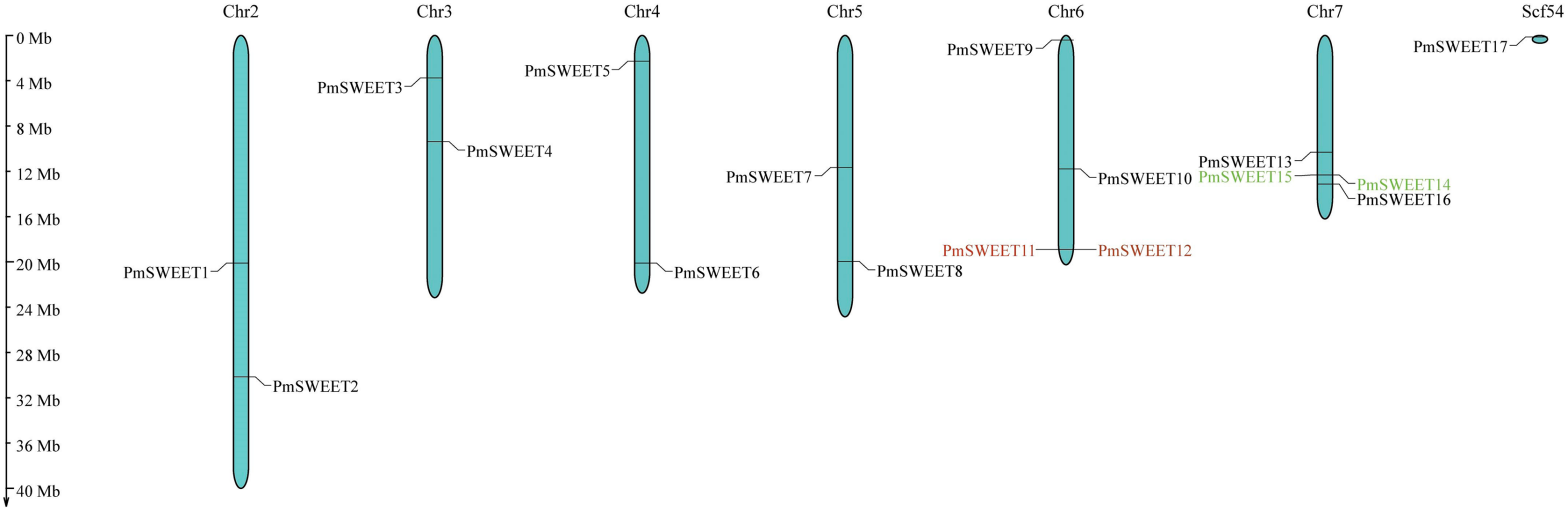
Schematic representations of the chromosomal location of the *PmSWEET* genes. The chromosome number is indicated on the top of each chromosome and/or scaffold. Scf54 indicates scaffold54. Green and red gene names indicate the two identified tandem duplicated gene pairs.

According to gene location information, all 17 *PmSWEETs* were mapped, showing that 16 *PmSWEETs* were located on chromosomes, and one *PmSWEET* gene was located on scaffold54 (Fig. 3). *PmSWEET* genes were mostly distributed on chromosomes 6 and 7, which both contained four *PmSWEET* genes. Two genes each were distributed on chromosomes 2, 3, 4 and 5. *PmSWEET11* and *PmSWEET12* as well as the *PmSWEET14* and *PmSWEET15* pair were clustered into two tandem duplication events on chromosomes 6 and 7, respectively. Based on the above results, some *PmSWEETs* gene family members were putatively generated by gene tandem duplication.

### Segmental duplication and synteny of the *PmSWEET* gene family

**Figure 4:**
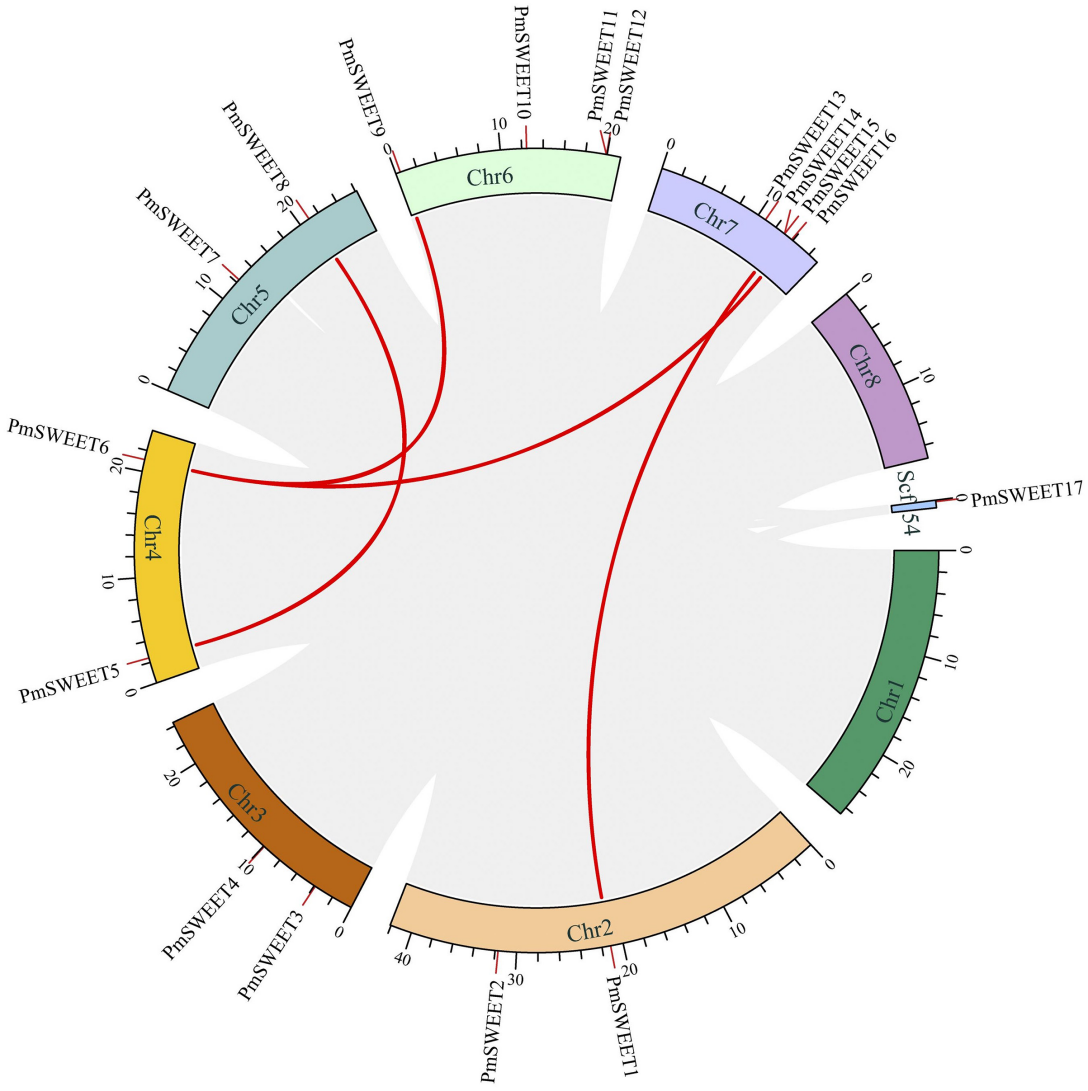
The Circos figure for *PmSWEET* segmental duplication links. The red lines indicate segmented duplicated gene pairs.

Synteny analysis of *PmSWEETs* was performed using the Circos program of TBtools, four segmental duplication events, including *PmSWEET1*/*PmSWEET14*, *PmSWEET5*/*PmSWEET8*, *PmSWEET6*/*PmSWEET9* and *PmSWEET6*/*PmSWEET16* were detected, and further, each gene pair was located on a different chromosome, as shown with red lines in Fig. 4. This finding strongly suggests that some *PmSWEETs* were likely generated by gene segmental duplication. In addition, the selection pressure and divergence time of the duplication events were estimated by the Ka (nonsynonymous) and Ks (synonymous) substitution ratio. In the evolutionary process, the Ka/Ks ratio >1 indicates positive selection (adaptive evolution), a ratio = 1 indicates neutral evolution (drift), and a ratio <1 indicates negative selection (conservation). Only one pair of segmentally duplicated *PmSWEETs*, namely *PmSWEET6* and *PmSWEET9*, had a Ka/Ks ratio of 0.45, which was significant, and indicated a synonymous change that has been selected during plant genome evolution. The differentiation period of the *PmSWEET6* and *PmSWEET9* gene pair was approximately 55.34 to 136.07 Mya.

**Figure 5:**
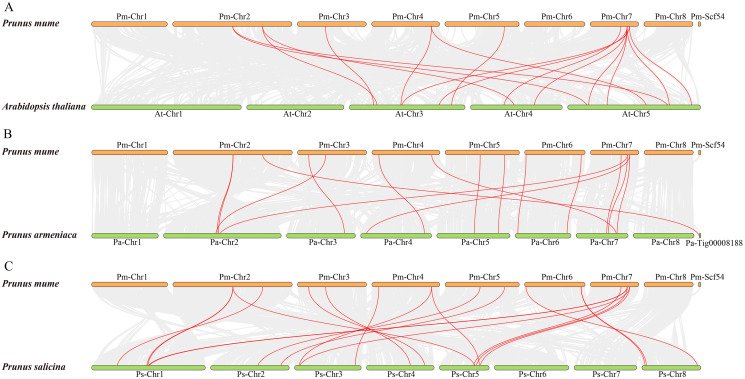
Synteny of *SWEET* genes in different genomes of *P. mume*, *A. thaliana*, *P. armeniaca* and *P. salicina*. (A) Synteny of *PmSWEET* and *AtSWEET* gene pairs. (B) Synteny of *PmSWEET* and *PaSWEET* gene pairs. (C) Synteny of *PmSWEET* and *PsSWEET* gene pairs.

To further examine the specific retention of *PmSWEETs*, their collinearity relationship with *AtSWEETs*, *PaSWEETs*, and *PsSWEETs* were detected using the MCScanX procedure of TBtools. A total of 16 homologous gene pairs were detected in *P. mume* and *A. thaliana*. Similarly, 16 pairs of homologous genes between *P. mume* and *P. armeniaca* and 20 between *P. mume* and *P. salicina* were detected (Fig. 5; Table S5). The collinear complexity of *P. mume* with *P. salicina* was much higher than that with *P. armeniaca* and *A. thaliana*. These results suggested that *P. mume* was relatively distantly related to *A. thaliana* and *P. armeniaca*, but is more closely related to *P. salicina*.

### Prediction analysis of *Cis*-acting elements within *PmSWEETs* gene promoters

**Figure 6:**
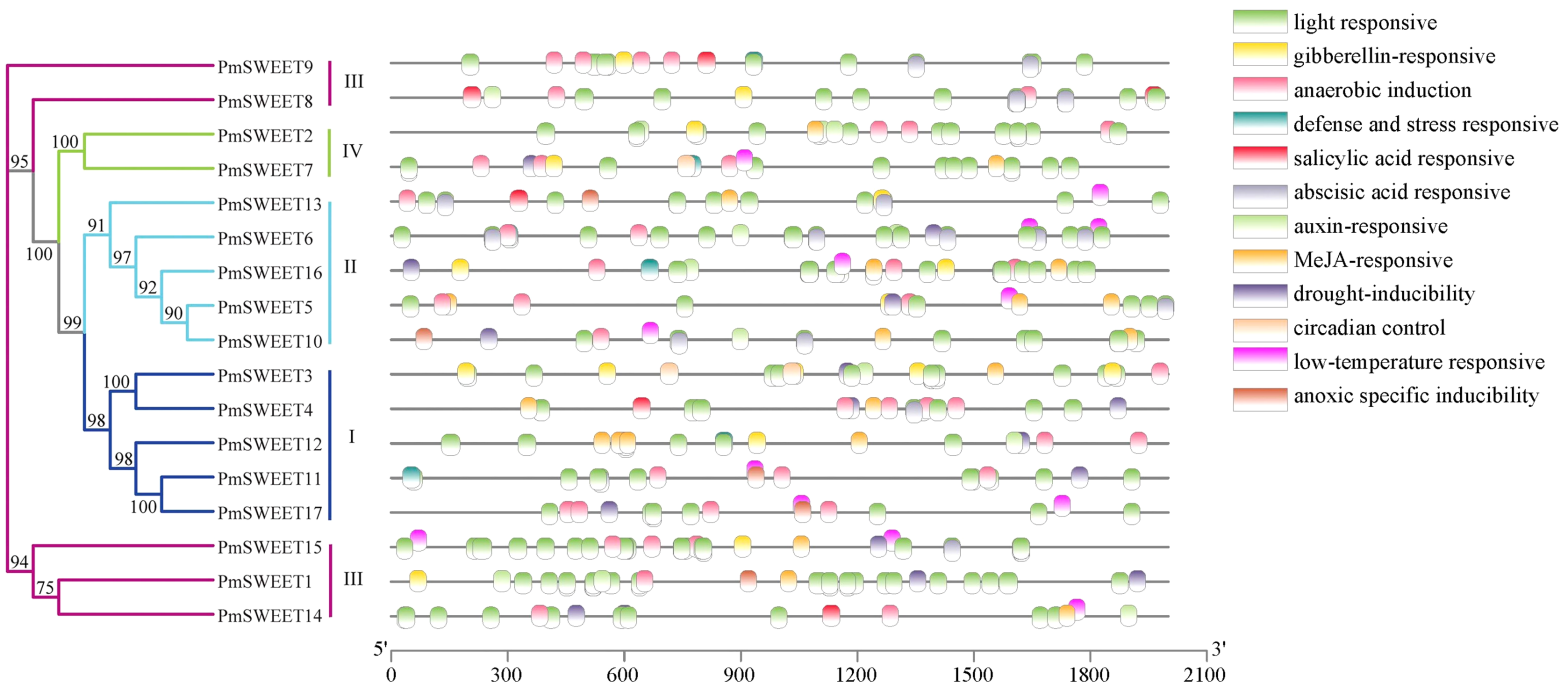
Predicted *cis*-elements responding to plant growth regulation, hormone response, and stress response present in *PmSWEET* gene promoters. Different colored boxes represent different elements and their positions in each *PmSWEET* promoter. The *SWEET* genes are classified into four clades, and blue, indigo, purple red, and green represent Clades I, II, III, and IV, respectively.

To further investigate the possible regulatory mechanism of *PmSWEETs* in the process of growth or in plant defence mechanisms, in particular the response of a plant to abiotic stresses such as low temperature, we submitted the 2.0 kb upstream sequence from the translation start site of each *PmSWEET* gene to the PlantCARE database to search for the presence of specific *cis*-elements. The *PmSWEET* promoters comprised several conserved regulatory elements that respond to plant hormones and environmental stress, and 12 of these were analyzed further (Fig. 6; Table S6). Elements related to light response, anaerobic induction, and ABA response were widespread in the promoter areas of 17, 17 and 16 members of the *P. mume SWEET* gene family, respectively. According to the regulatory elements in their promoters, 14, 12, 11, 10, and 9 *P. mume SWEET* gene family members were sensitive to drought inducibility, MeJA, gibberellin, low temperatures and auxin, respectively. By combining these findings with the results of phylogenetic analysis, it was found that gene members of the same clade had similar *cis*-elements. These results indicated that *PmSWEET* genes were involved in the regulatory mechanisms of various stress responses.

### Expression pattern analysis of *PmSWEETs*

**Figure 7:**
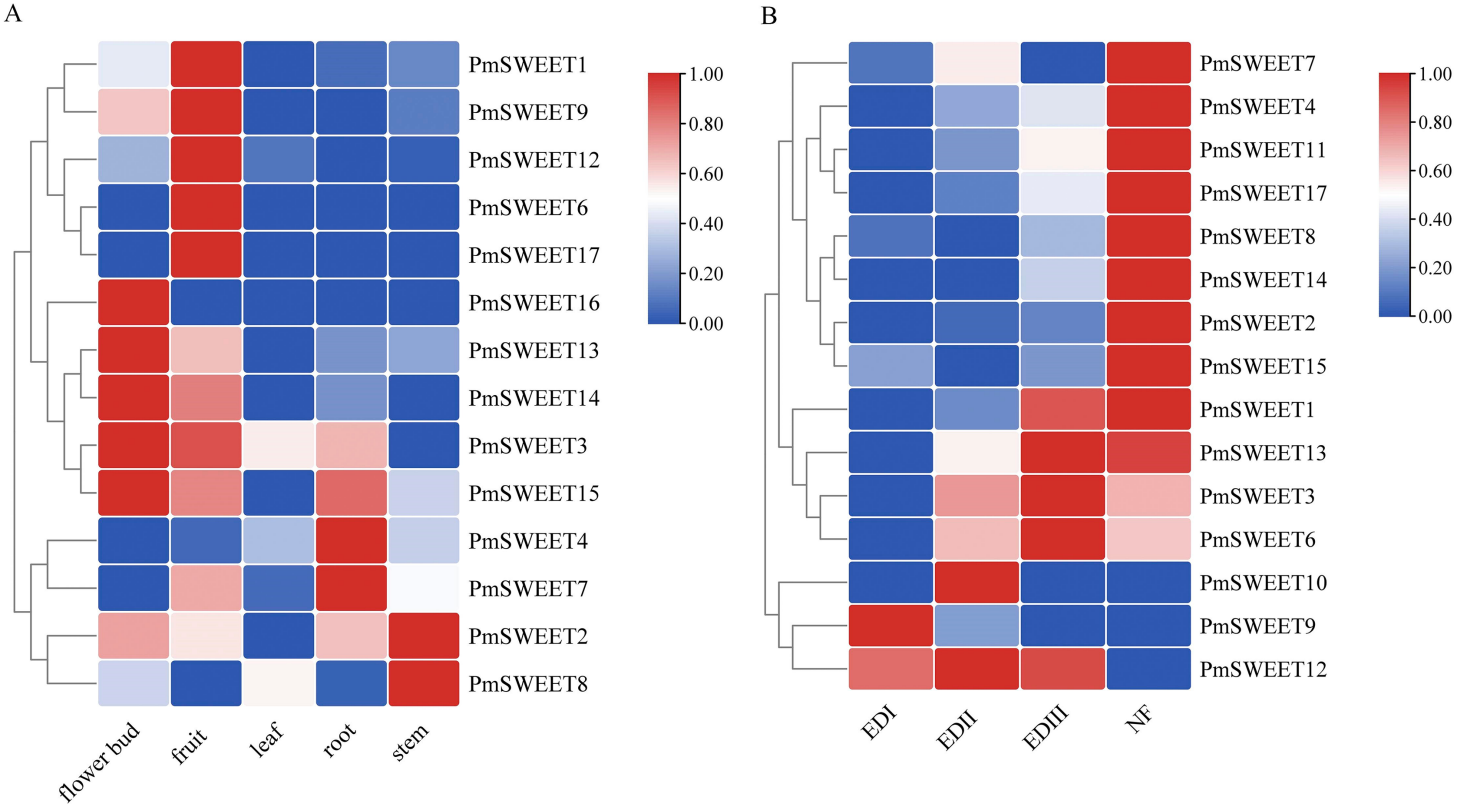
Expression profiles of *PmSWEET* genes in different tissues and different flower buds stage. (A) Expression profiles of *PmSWEETs* in different tissues. (B) Expression profiles of *PmSWEETs* in the flower bud during dormancy. EDI, endo-dormancy I, November; EDII, endo-dormancy II, December; EDIII, endo-dormancy III, January; NF, natural flush, February. A 2-based log function conversion is performed on the expression amount, and then normalized by row using min-max method. The color scale on the right of the heat map refers to relative expression level, and the color gradient from blue to red shows an increasing expression level.

To investigate the role of *PmSWEETs* in development and response to low temperature, the expression patterns of family members in the roots, stems, leaves, flower buds, fruits (Zhang et al., 2012) and flower buds of different stages of dormancy (Zhang et al., 2018), were examined based on our RNA-seq dataset, and their RPKM values are shown in Tables S7 and S8. As illustrated in Fig. 7A, 14 of the *PmSWEET* genes were expressed in at least one tissue, whereas RNA-seq failed to detect the expression of three family members, namely *PmSWEET5*, *PmSWEET10* and *PmSWEET11*. Among them, five *PmSWEETs* presented relatively higher expression levels in fruits (*PmSWEET1*, *PmSWEET6*, *PmSWEET9*, *PmSWEET12* and *PmSWEET17*) and flower buds (*PmSWEET3*, *PmSWEET13*, *PmSWEET14*, *PmSWEET15* and *PmSWEET16*). Two *PmSWEETs* showed higher expression levels in roots (*PmSWEET4* and *PmSWEET7*) and stems (*PmSWEET2* and *PmSWEET8*). Additionally, genes *PmSWEET2*, *PmSWEET3*, *PmSWEET4*, *PmSWEET7*, *PmSWEET8*, *PmSWEET12* and *PmSWEET13* were expressed in leaves, but their expression levels were low.

Most *PmSWEETs* were expressed during the flower bud dormancy period (except *PmSWEET5* and *PmSWEET16*) as well as being expressed at specific stages of development (Fig. 7B). Ten *PmSWEET* genes exhibited specifically higher expressions in the natural flush (NF) stage (February), *PmSWEET9* was preferentially expressed in the endo-dormancy I (EDI) stage (November), *PmSWEET10* and *PmSWEET12* showed the highest level of expression in the endo-dormancy II (EDII) stage (December); and *PmSWEET1*, *PmSWEET3*, *PmSWEET6*, *PmSWEET12* and *PmSWEET13* showed upregulated expression in the endo-dormancy III (EDIII) stage (January). Among these upregulated genes, eight *PmSWEETs* (*PmSWEET6*, *PmSWEET7*, *PmSWEET10*, *PmSWEET11*, *PmSWEET13*, *PmSWEET14*, *PmSWEET15* and *PmSWEET17*) (Table S6) contained low temperature response elements within their putative promoter regions.

**Figure 8:**
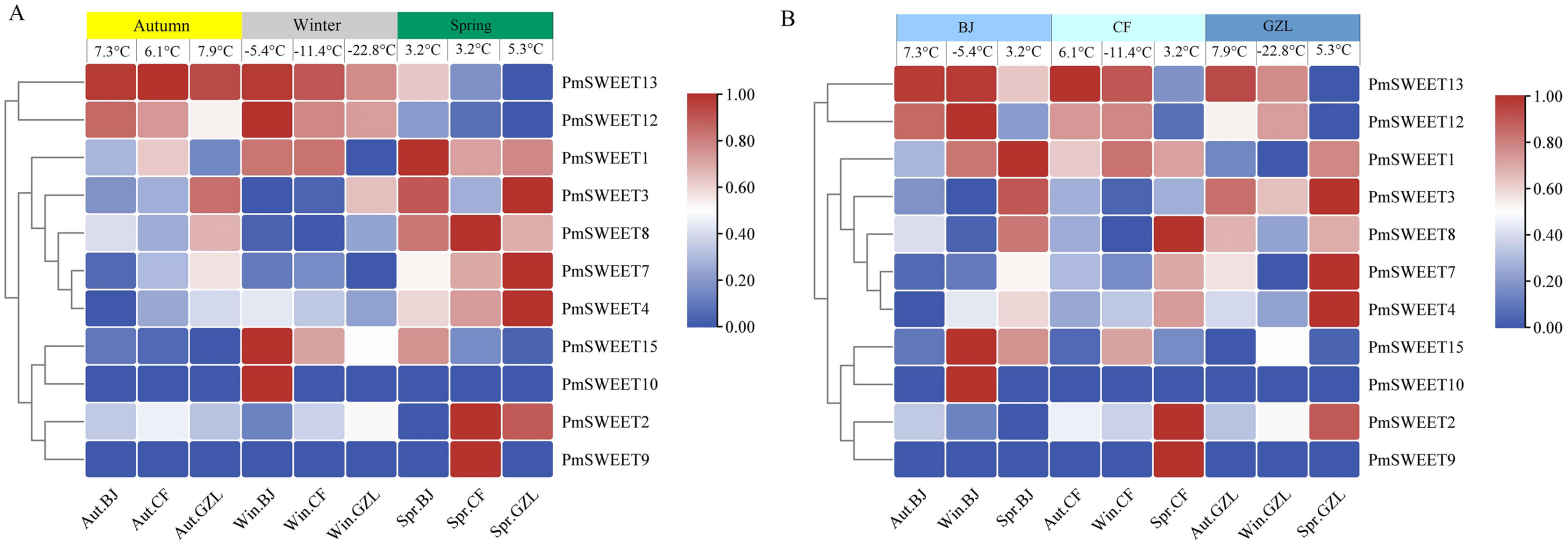
Expression profiles of *PmSWEETs* in stems in different seasons and regions. (A) Expression profiles of *PmSWEETs* in stems of ‘Songchun’ in different regions (Beijing, Chifeng and Gongzhuling) and seasons (autumn, winter and spring). (B) Comparison of differential expression profiles of stems in Beijing, Chifeng and Gongzhuling during different seasons. A 2-based log function conversion is performed on the expression amount, and then normalized by row using min-max method. The color scale on the right of the heat map refers to relative expression level, and the color gradient from blue to red shows an increasing expression level. Aut, Autumn; Win, Winter; Spr, Spring; BJ, Beijing; CF, Chifeng; GZL, Gongzhuling.

To further investigate the expression patterns of *PmSWEETs* under cold exposure, we analyzed the stems of the cold-tolerant cultivar *P. mume* ‘Songchun’ at three geographically distinct locations (Jiang, 2020), and their FPKM values are displayed in Table S9. The expression of six *PmSWEET* genes (*PmSWEET5*, *PmSWEET6*, *PmSWEET11*, *PmSWEET14*, *PmSWEET16* and *PmSWEET17*) was not detected. Among the other 11 *PmSWEET* genes, seven *PmSWEETs* (*PmSWEET1*, *PmSWEET2*, *PmSWEET3*, *PmSWEET4*, *PmSWEET7*, *PmSWEET8* and *PmSWEET9*) showed higher expression in spring (3.2~5.3 °C). *PmSWEET13* expression was upregulated in autumn (6.1~7.9 °C) and winter in Beijing (–5.4 °C) and Chifeng (–11.4 °C), but downregulated in spring; the expression levels of *PmSWEET10*, *PmSWEET12* and *PmSWEET15* increased significantly in winter in Beijing (–5.4 °C) (Fig. 8A). Among these genes with upregulated expression, four *PmSWEETs* (*PmSWEET7*, *PmSWEET10*, *PmSWEET13* and *PmSWEET15*) (Table S6) contained low-temperature response elements within their putative promoter regions. To compare the expression patterns of *PmSWEETs* during different times of the year, another heatmap was generated (Fig. 8B). As shown in Fig. 8B, *PmSWEETs* expression in the material sourced from the locations, Chifeng and Gongzhuling showed similar expression patterns at the same time of the year, while *PmSWEETs* expressed for the material sourced from the Beijing location showed higher expression in winter (Fig. 8B). This may be related to the latitude of the three geographical sampling locations, Gongzhuling has the highest latitude, followed by Chifeng and Beijing. There is little difference between the temperature in autumn and spring in the three sampling locations, however there is considerable difference in the winter temperature. In winter, the temperature in Beijing (–5.4 °C) is higher than that in the other two sampling locations (Gongzhuling is –22.8 °C, Chifeng is –11.4 °C), which may be the temperature that is required to induce the expression of some *P. mume SWEET* gene family members.

### Expression patterns of *P. mume SWEETs* under cold treatment

**Figure 9:**
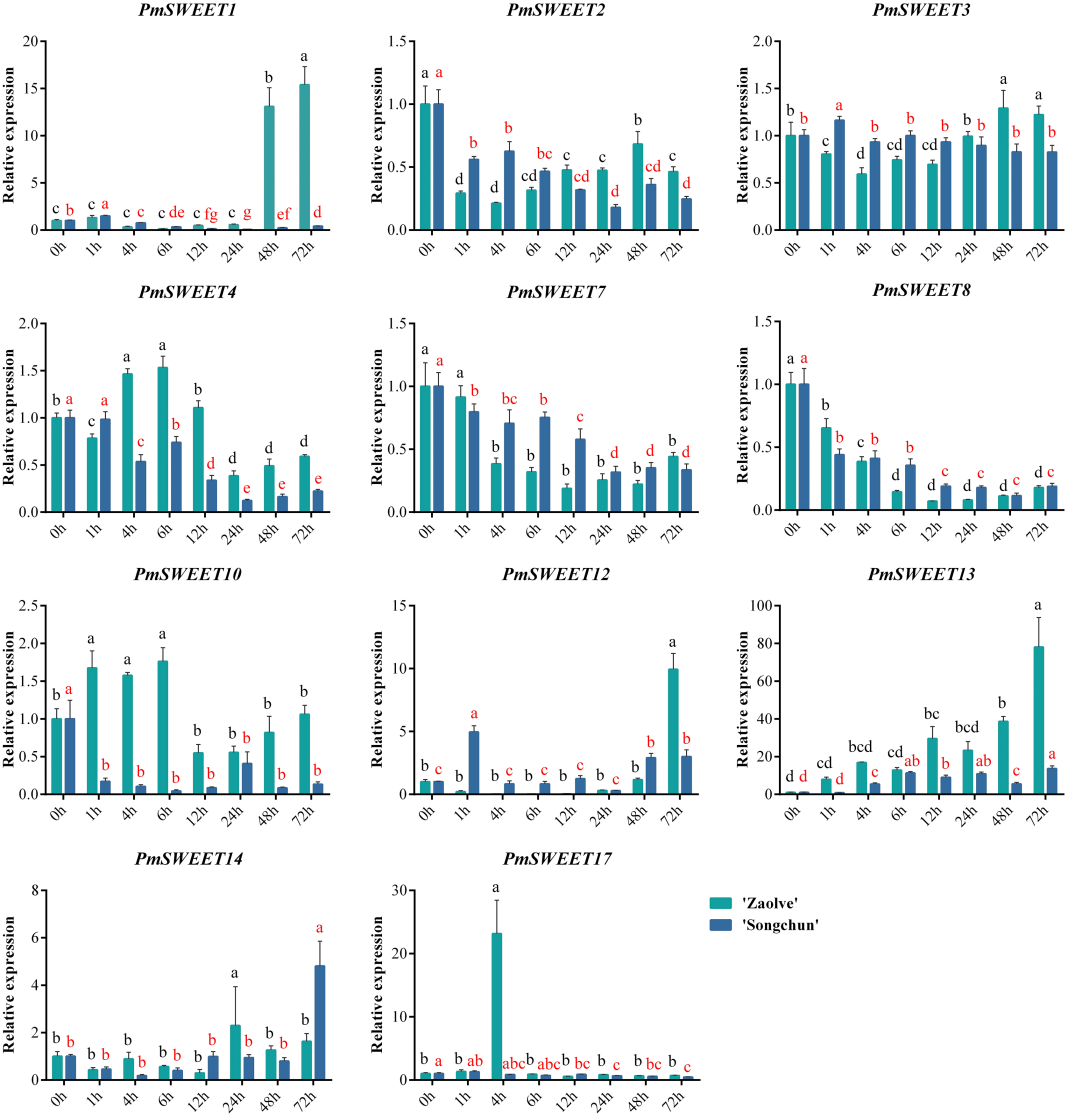
Expression patterns of 11 *PmSWEET* genes under low temperature treatments. The relative quantification method (2^–ΔΔCt^) was used to evaluate the transcript levels of 11 *PmSWEET* genes. Error bars are standard deviation of three biological replicates. The statistical analyses of ‘Zaolve’ and ‘Songchun’ were conducted independently using SPSS22.0, the one-way ANOVA analysis of variance was calculated by least significant difference (LSD) and Student–Newman–Keuls test, diﬀerent letters above the bars indicate significant diﬀerences (*p* = 0.05). Black letters indicate ‘Zaolve’, red letters indicate ‘Songchun’. GraphPad Prism6 software was used to draw the diagram.

To investigate the role of *PmSWEETs* in response to cold stress, the expression patterns under imposed stress treatment temperature of 4 °C for 0, 1, 4, 6, 12, 24, 48 and 72 h were examined by qRT–PCR using the cold-sensitive cultivar ‘Zaolve’ and the cold-tolerant cultivar ‘Songchun’. We performed a qRT–PCR assay on the 17 identified *P. mume SWEETs*, but the expression of only 11 *PmSWEETs* was detectable by this approach, while the remaining 6 *PmSWEETs* (*PmSWEET5*, *PmSWEET6*, *PmSWEET9*, *PmSWEET11*, *PmSWEET15* and *PmSWEET16*) were not detected, a finding that is consistent with the transcriptome data (Figs. 7 and 8). As displayed in Fig. 9, the changes in expression levels of the 11 *SWEET* genes in the two cultivars differed during the imposed cold stress treatment period. In the two assessed cultivars, the expression of three genes, *PmSWEET2*, *PmSWEET7* and *PmSWEET8*, was reduced. In addition, the expression of *PmSWEET13* was upregulated in both ‘Songchun’ and ‘Zaolve’, which rose approximately 11-fold after 6 h of cold treatment in ‘Songchun’, while rising approximately 9-fold after 1 h, and then increased nearly 80-fold after 72 h of cold treatment in ‘Zaolve’. One gene (*PmSWEET3*) changed only slightly in both ‘Songchun’ and ‘Zaolve’. Six genes (*PmSWEET1*, *PmSWEET4*, *PmSWEET10*, *PmSWEET12*, *PmSWEET14*, and *PmSWEET17*) exhibited different expression patterns in the two cultivars. Among these six genes, *PmSWEET1* and *PmSWEET12* were upregulated initially, then downregulated with increasing treatment duration in ‘Songchun’, while in ‘Zaolve’, there was no obvious change in the early treatment stages, but the expression of these two *PmSWEETs* increased considerably at 48 h and 72 h, respectively. *PmSWEET4* and *PmSWEET10* were dramatically downregulated in their level of expression with increased cold stress duration in ‘Songchun’, while the expression of these two *PmSWEETs* was upregulated within 6 h and then decreased with extended treatment in ‘Zaolve’. *PmSWEET14* expression did not show an obvious change across the early stages of treatment, but was rapidly upregulated at 72 h in ‘Songchun’, and at 24 h in ‘Zaolve’, and then the expression level of *PmSWEET14* decreased in ‘Zaolve’ with increasing treatment duration. The expression of *PmSWEET17* was increased during the early stages of treatment, but then decreased with increased treatment duration in ‘Songchun’, while it was highly expressed only at 4 h in ‘Zaolve’.

## Discussion

*SWEET* genes form a family of sugar transporters that play a role in the transportation of sugars, mainly sucrose, glucose and fructose (Chen et al., 2010, 2012; Feng & Frommer, 2015; Guo et al., 2014; Klemens et al., 2013; Le Hir et al., 2015), and due to this important role, *SWEETs* have been demonstrated to function in diverse physiological and biological processes in the growth and development of many plants as well as in the response of these plant species to biotic and abiotic factors (Lemoine et al., 2013; Li et al., 2017, 2018; Zhao et al., 2018). Previous studies have shown that *SWEETs* participate in cold stress responses in several plant species (Chardon et al., 2013; Klemens et al., 2013; Guo et al., 2014; Chong et al., 2014; Liu et al., 2016; Le Hir et al., 2015; Yue et al., 2015; Wang et al., 2018; Feng et al., 2015; Miao et al., 2017; Zhang et al., 2019). However, little is known about the potential roles of *SWEET* genes in the response of *P. mume* to cold stress. *P. mume* has a high ornamental value, and can blossom at lower temperatures; but different cultivars have different cold resistance, making it an ideal plant species for studying the mechanisms of how *SWEET* genes function in cold responses. Understanding the link between *SWEET* genes of *P. mume* and cold-resistance could provide insights into cold-resistance molecular breeding in the future. In this research, we identified a total of 17 *SWEET* genes in *P. mume*, the same number that is that present in *Arabidopsis*, and similar to the numbers in other species of *Prunus*, showing that *SWEET* genes are still relatively conserved in *Prunus*. The length of *Pm*SWEET proteins ranged from 105 aa to 580 aa, and this range provides diversity in the number of TMHs (2–7). *PmSWEETs*, except for *PmSWEET14*, have a theoretical pI larger than 8.0. As an important parameter of proteins, pI is determined by the relative contents of amino acid residues at different pH values, which affects the stability, activity and function of a protein (Gasteiger, 2005). The pI of *PmSWEET14* was not detected, which may be due to its short amino acid sequence.

By predicting TMH domains, we found that the number of TMHs encoded by *PmSWEET* genes ranged from 2 to 7 (Table 1). Fewer than seven TMHs in members of the *SWEET* gene family has also been reported previously in other plant species, including wheat (Gao et al., 2018; Gautam et al., 2019), walnut (Jiang et al., 2020), *Kentucky bluegrass* (Zhang, Niu & Ma, 2020) and soybean (Patil et al., 2015). To further validate the accuracy of our SWEET protein predictions, we submitted the protein sequence of each *Pm*SWEET to the NCBI-CDD and SMART online tools to predict their conserved domains, and it was found that each assessed family member contained the MtN3_slv domain, and therefore, belonged to the *SWEET* gene family. This result also indicated that duplication and fusion, or genetic loss may have occurred to individual *SWEET* gene loci as part of the evolution of the *P. mume* genome. Similar to the case in other plant species (Chen et al., 2010; Yuan & Wang, 2013; Patil et al., 2015), *PmSWEETs* can be classified into four clades, and the number of *SWEET* genes members from 11 plant species ordered into Clade III was larger than that in the other three clades (Fig. 1), suggesting that Clade III may have expanded during genome evolution. Conserved motif analysis indicated that some special motifs only reside in some *PmSWEET* gene family members. For instance, motif 8 was only present in *PmSWEET11* and *PmSWEET17*; and motif 10 was only present in *PmSWEET3* and *PmSWEET15*. These results are consistent with those of other plant species, such as *Arabidopsis* (Chen et al., 2010), rice (Yuan & Wang, 2013), banana (Miao et al., 2017) and wheat (Gautam et al., 2019). Together, these studies have demonstrated that gene structural diversity and conserved protein motif divergence has performed a key role in the evolution of the *SWEET* gene family (Xu et al., 2012). More specifically, specific *PmSWEETs* harbored unique conserved motifs, implying that such family members may be responsible for the functional diversity of the *P. mume SWEET* gene family.

Gene duplication, including tandem and segmental duplication events, is the origin of gene family expansion as part of genome evolution in plants (Cannon et al., 2004; Ganko, Meyers & Vision, 2007). In this study, two pairs of *PmSWEETs* were detected as tandem duplications, and four pairs of *PmSWEETs* were identified to be the result of segmental duplications. This finding is consistent with those of other studies on *SWEET* duplication, including segmental and tandem duplications (Feng et al., 2015; Miao et al., 2017; Gao et al., 2018; Jiang et al., 2020).

The *cis*-elements in the promoter of a gene play an essential role in the regulation of gene expression, and therefore, gene function. All *PmSWEETs* contain at least one light-responsive and anaerobically induced *cis*-element, suggesting that these two elements have an essential role in regulating *PmSWEET* gene expression. Moreover, 10 *PmSWEETs* contained one or more low-temperature responsive *cis*-elements (Table S6), indicating that these *PmSWEETs* may play important roles in the response of a *PmSWEET* gene to cold stress. However, the exact regulatory role directed by these *cis*-elements in *P. mume* requires further research.

Studies have shown that under low-temperature stress, the soluble sugar content in plants increases, and sugar transporters maintain the balance of osmotic potential through the balance and distribution of sugar, thus improving the cold tolerance of plants (Yamada et al., 2010). Numerous studies have also verified that *SWEETs* are involved in maintaining sugar homeostasis in plant organs and promoting plant adaptation to low temperatures (Seo et al., 2011; Chardon et al., 2013; Klemens et al., 2013; Chandran, 2015; Le Hir et al., 2015; Miao et al., 2017; Wang et al., 2018; Zhang et al., 2019; Zhang, Niu & Ma, 2020). Transcriptome analysis showed that *PmSWEETs* were differentially expressed in different tissues and during dormancy release and cold acclimation. *PmSWEET5* expression was not detected in any tissue/organ that was assessed in this study, indicating that its expression may be highly varietal, spatially and temporally specific. Some *PmSWEETs* had specific expression patterns in different organs (Fig. 7A). For example, *PmSWEET10* expression was detected only in ‘Zaolve’ flower buds at dormancy (stage EDII) and ‘Songchun’ stems of those *P. mume* plants taken from the Beijing winter sampling site. Furthermore, *PmSWEET16* expression was detected only in *P. mume* flower buds, which indicates that these two *PmSWEETs* are expressed only in specific tissues, cultivars, or environmental conditions, with such organ-specific expression previously observed in wheat (Gao et al., 2018; Gautam et al., 2019), walnut (Jiang et al., 2020), tea (Wang et al., 2018) and cabbage (Zhang et al., 2019). *AtSWEET5*, the homologue of *PmSWEET10* and *PmSWEET16*, plays a key role in seed germination, and is expressed at different stages of pollen development (Engel, Holmes-Davis & McCormick, 2005). The results from expression studies of different organs indicate a role for *PmSWEET10* and *PmSWEET16* in pollen development, suggesting they might play a similar role to *AtSWEET5*. *PmSWEET1*, *PmSWEET*6, *PmSWEET9*, *PmSWEET12* and *PmSWEET17* were strongly expressed in fruit, to indicate that the proteins encoded for by these *PmSWEET* genes may regulate sugar allocation during fruit ripening. Such specific, and high levels of expression of *SWEETs* in fruits has also been found in pineapple (Guo et al., 2018), sweet orange (Zheng et al., 2014) and apple (Zhen et al., 2018), findings which collectively infer that SWEET proteins likely mediate an important role in fruit development and ripening. *PmSWEET4* (Clade I) and *PmSWEET7* (Clade IV) were strongly expressed in roots, this results had similar expression patterns to previous studies, those being that Clade IV *SWEET* genes are highly expressed in the root cortex and encode for proteins that function as fructose-specific uniporters in the root vacuole membrane (Guo et al., 2014).

The present results also show that most of the *PmSWEET* genes are expressed more strongly at different endo-dormancy stages in flower bud and fruit tissues than in other tissues and that these genes are differentially expressed during flower development (Figs. 7A and 7B). Together, these results suggest that the *P. mume SWEET* gene family is closely associated with reproductive development and that different genes are specifically involved during different developmental stages. In rice, *Arabidopsis* and soybean, the expression of *SWEET* genes is also higher in reproductive tissues than in other tissues (Yuan et al., 2014; Patil et al., 2015). *PmSWEETs* also have different expression levels during dormancy release in flower buds (from November to February). Thus, we speculate that these *PmSWEETs* may participate in the cold reaction at low temperatures to protect the flower bud. In addition, some *PmSWEETs* were expressed more highly at colder temperatures in the spring (3.2~5.3 °C) and at approximately –5 °C in the winter (Fig. 8A). Together, this finding putatively suggests that these two temperatures may trigger their cold stress response and increase *PmSWEET* expression to reduce stress injury.

The qRT–PCR analysis suggested that six of 17 *PmSWEET* genes (*PmSWEET5*, *PmSWEET6*, *PmSWEET9*, *PmSWEET11*, *PmSWEET15*, and *PmSWEET16*) were not expressed in the stem, which was consistent with the transcriptome data. *PmSWEETs* were activated by low temperature (4 °C) and increased or decreased in expression with the extension of treatment time (Fig. 9). The expression levels of five *PmSWEETs* (*PmSWEET2*, *PmSWEET4*, *PmSWEET7*, *PmSWEET8*, and *PmSWEET10*) in ‘Songchun’ and three *PmSWEETs* (*PmSWEET2*, *PmSWEET7*, and *PmSWEET8*) in ‘Zaolve’ decreased with increasing treatment duration (Fig. 9), which suggested that these genes might be negatively regulated by low temperatures and result in increased cold sensitivity. The expression levels of two *PmSWEETs* (*PmSWEET13* and *PmSWEET14*) in ‘Songchun’ and three *PmSWEETs* (*PmSWEET1*, *PmSWEET12*, and *PmSWEET13*) in ‘Zaolve’ increased with prolonged treatment (Fig. 9), which suggested that these genes might be positively regulated by cold stress responses and increase the cold sensitivity of *P. mume*. The discrepancy in expression patterns between *PmSWEET1*, *PmSWEET4*, *PmSWEET10*, *PmSWEET12*, *PmSWEET14* and *PmSWEET17* is potentially due to genetic differences between ‘Songchun’ and ‘Zaolve’.

## Conclusions

In summary, our study is the first to perform genome-wide identification and characterization of *SWEETs* in *P. mume*, including chromosomal location, duplicated gene identification, gene structure analysis, phylogenetic relationships and conserved motifs. In addition, the expression profiles of the *PmSWEET* genes in different tissues and geographic locations were also examined based on the RNA-seq data. Furthermore, the expression profiles of these *PmSWEET* genes under cold stress conditions were analyzed by qRT–PCR assay. Our results could provide important information for further research on the biological functions of *PmSWEETs*.

## Supplemental Information

10.7717/peerj.13273/supp-1 Supplemental Information 1 Supplemental tables.

10.7717/peerj.13273/supp-2 Supplemental Information 2 Schematic representation of PmSWEET proteins. Colored boxes indicate TMs. The IBS1.0 software was used to drawing diagram (Liu et al., 2015).

10.7717/peerj.13273/supp-3 Supplemental Information 3 Phylogenetic trees of *Arabidopsis thaliana*, *Prunus mume* and Rice.

10.7717/peerj.13273/supp-4 Supplemental Information 4 Schematic diagram of PmSWEET protein motifs.

10.7717/peerj.13273/supp-5 Supplemental Information 5 Protein sequences of *P. mume*.

10.7717/peerj.13273/supp-6 Supplemental Information 6 Domain architecture of *PmSWEETs*.

10.7717/peerj.13273/supp-7 Supplemental Information 7 ’Zaolve’ qRTPCR raw data. Expression analyses of 11 *PmSWEETs* in *P. mume* ’Zaolve’ exposed to 4 °C for different times (0/1/4/6/12/24/48/72 h), where 0 h indicates control.

10.7717/peerj.13273/supp-8 Supplemental Information 8 ’Songchun’ qRTPCR raw data. Expression analyses of 11 *PmSWEETs* in *P. mume* ’Songchun’ exposed to 4 °C for different times (0/1/4/6/12/24/48/72 h), where 0 h indicates control.

## Additional Information and Declarations

### Data Availability

The following information was supplied regarding data availability: The raw dates are available in the Supplemental Files.
