# Supplementary material for: Genome-wide identification of the SWEET gene family mediating the cold stress response in Prunus mume
Source: PeerJ. 2022 May 3;10:e13273. doi: 10.7717/peerj.13273 (PMC9074862; doi:10.7717/peerj.13273)
Supplement: Supplemental Information 2 — Colored boxes indicate TMs. The IBS1.0 software was used to drawing diagram (Liu et al., 2015). [file peerj-10-13273-s002.pdf]

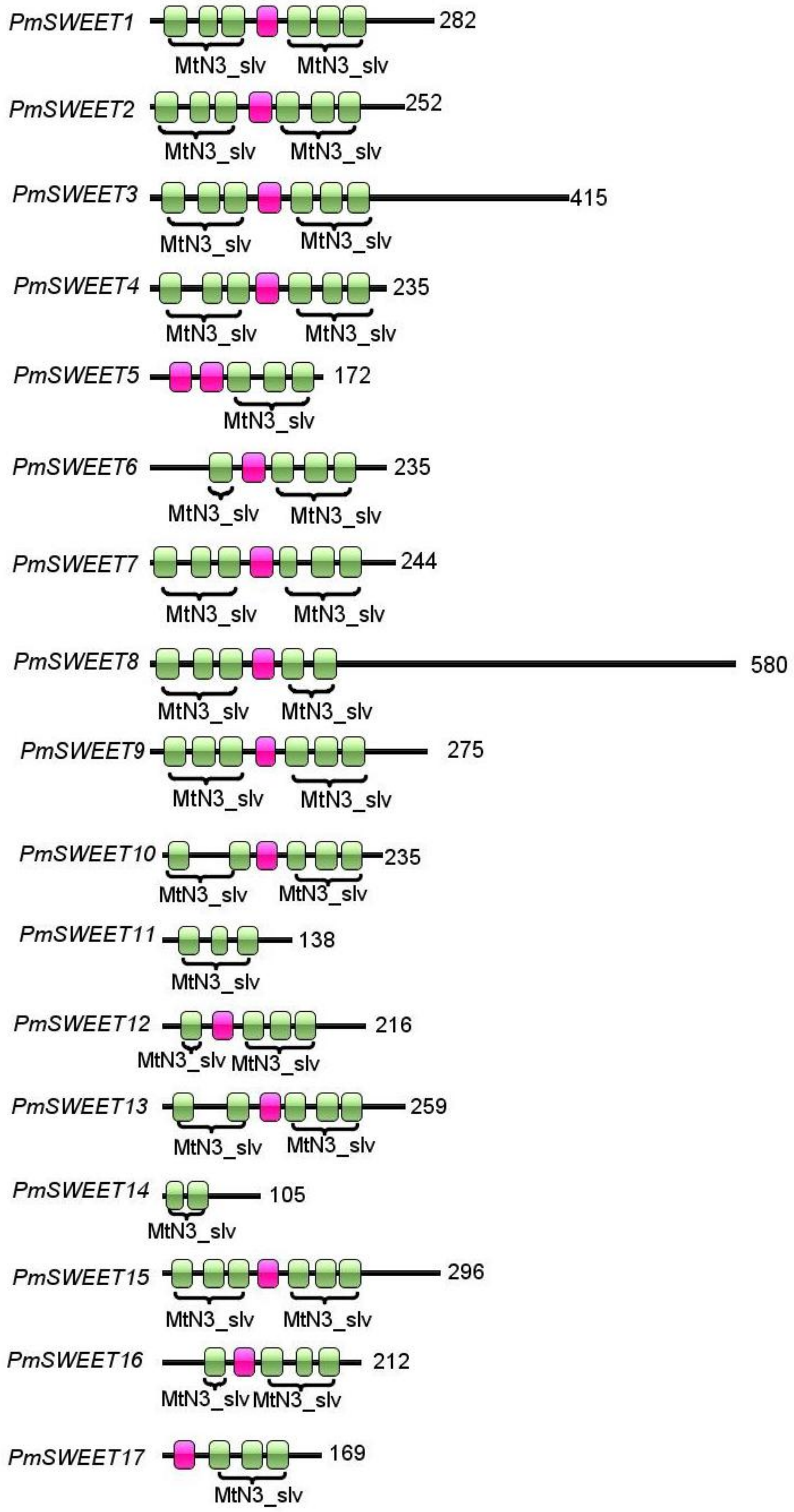

Supplementary Figure 1. Schematic representation of *Pm*SWEET proteins. Colored boxes indicate TMs. The IBS1.0 software was used to drawing diagram (Liu et al., 2015).

Liu, W., Xie, Y., Ma, J., Luo X., Nie, P., Zuo, Z., et al. (2015). IBS: an illustrator for the presentation and visualization of biological sequences. *Bioinformatics.* 31(20): 3359-3361.
