# Supplementary figures and images for: Genome-wide identification of the SWEET gene family mediating the cold stress response in Prunus mume
Source: PeerJ. 2022 May 3;10:e13273. doi: 10.7717/peerj.13273 (PMC9074862; doi:10.7717/peerj.13273)

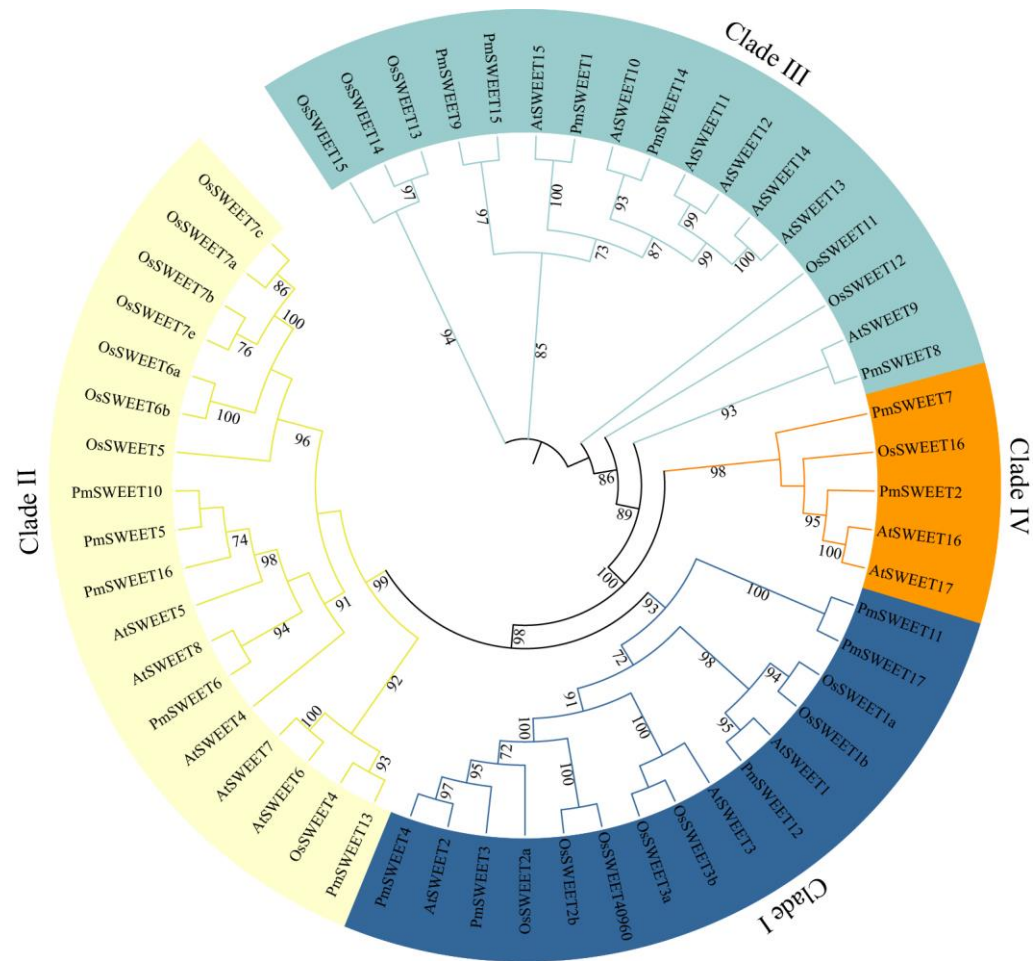

Supplementary Figure 2. Phylogenetic trees of *Arabidopsis thaliana*, *Prunus mume* and rice

Supplement: Supplemental Information 3 [file peerj-10-13273-s003.pdf]
