# Supplementary material for: Genome-wide identification of the SWEET gene family mediating the cold stress response in Prunus mume
Source: PeerJ. 2022 May 3;10:e13273. doi: 10.7717/peerj.13273 (PMC9074862; doi:10.7717/peerj.13273)
Supplement: Supplemental Information 4 [file peerj-10-13273-s004.pdf]

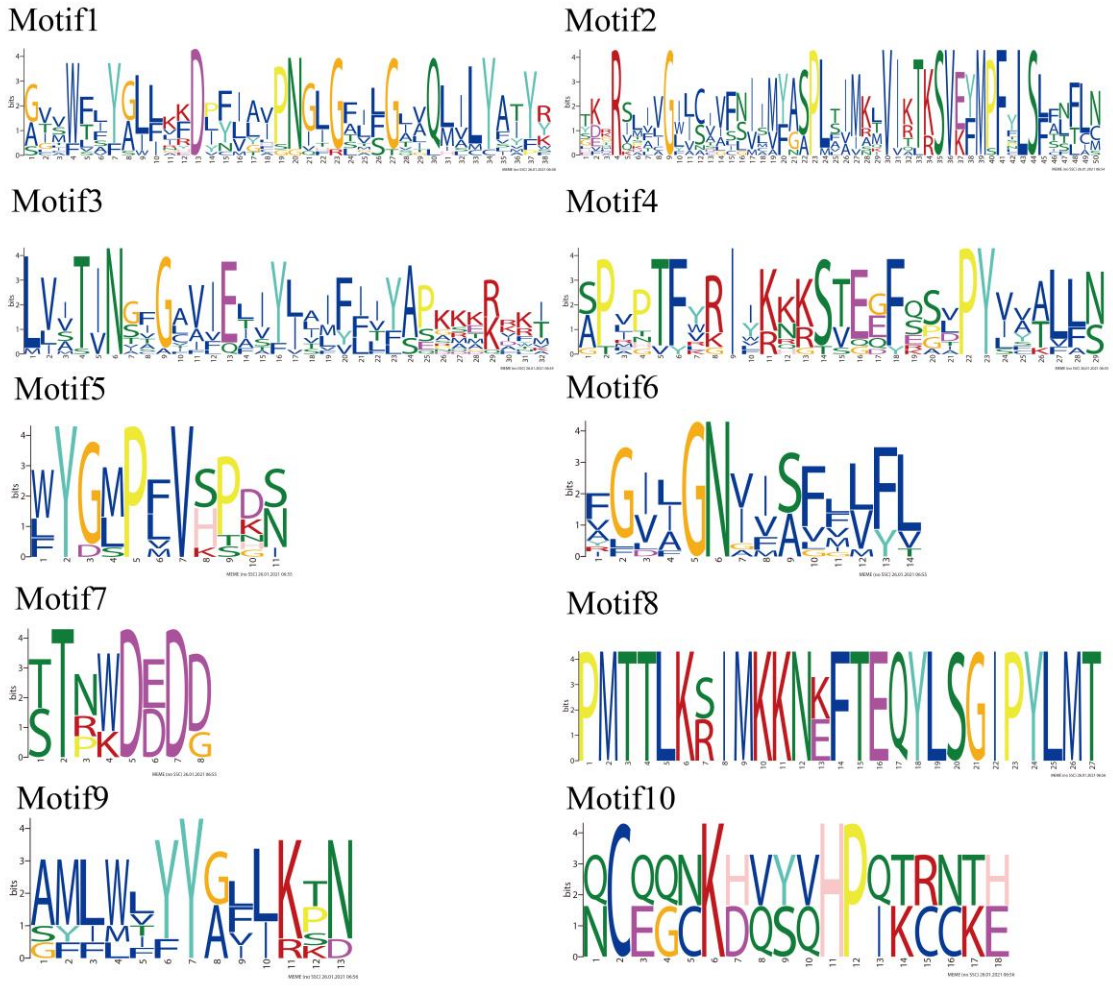

Supplementary Figure 3. Schematic diagram of *Pm*SWEET protein motifs. The bit score is proportional to the frequency of the corresponding amino acid at each position.
