# Supplementary material for: Genome-wide identification of the SWEET gene family mediating the cold stress response in Prunus mume
Source: PeerJ. 2022 May 3;10:e13273. doi: 10.7717/peerj.13273 (PMC9074862; doi:10.7717/peerj.13273)
Supplement: Supplemental Information 5 [file peerj-10-13273-s005.docx]

**Protein sequences of *P. mume***

**>Pm007067 (PmSWEET1) locus=Pm2:21184396:21186332:- [translate_table: standard]**

MGALADSHHPWAFTFGILGNVISFLVYLAPMPTFYGIYKKKSTQGFQSVP

YLVALFSGMLWFYYALLKKNAMLLITINSFGTVIETIYIVMFIFYAPKDA

RKFTLKLFGFMNVGLFCSILVLSHFAVRSEYRVPVLGWINVAISVIVFAA

PLSIVAQVIRTRSVEFMPFSLSFFLTLSAVMWFSYGLFLKDICIAIPNVL

GFILGLLQMLLYAIYRNREQVIIEDDEKKIPAAAAADQHVKNVVGLTTLA

TSEVHPVDPPPHDHDKSVEVDSGSHTAAASCA

**>Pm008206 (PmSWEET2) locus=Pm2:31718730:31721555:- [translate_table: standard]**

MEGLILFIGVIGNIISVLMFLAPVGTFWRIVKHRSTEDFESLPYVCTFLN

SFLWTYYGIIRPNGGFLVATVNGFGVVVEIIYLILFLVYAPAKMRAKTAI

LIGTLDVGFLAAAILATWLALQGKTRIAALGFICAGLNILMYGSPLVAMK

TVITTKSVEYMPFFLSFFFFLNGGVWTLYAWLIRDVFLGVPNGIGFLLGT

TQLVLYAIYRNAKPADNISSGLLEEGRQHEPLISPSSATLSHQKNGEILE

TT

**>Pm010330 (PmSWEET3) locus=Pm3:3891190:3895205:- [translate_table: standard]**

MLSTGLSSVYSGFSTAAGIAGNIFAFVLFVSPLPTFKRIIRNKSTEQFSG

LPYIYALLNCLICLWYGMPVVKTGIILVATVNSFGAVFQLVYLSIFITYA

ERATKLRMLGLLGAVAVIFALVVFMSLGILEYDDRQTFVGYLSVASLISM

FASPLFIIKLVIKTRSVEFMPFNLSFATFLMSISFSAYGIFKEDPFLYIP

NGIGTILGLVQLALYSYYRKISGEDSREPLTLYRLEDMAIGIYAVAGKGV

IAKEQYQILLKFYQKETDKLMQNLVLFKAVYGLLGWYLLYSAADGSHSRI

APDGDPLSSCKVNVPKTKKTYCKSKECKKHTLHKVTQYKTGKASIAAQGK

RRYDRKQSGYGGQTKPVFHKKAKTTKKIVLRLQCQGCKHVSQHPIKRCKH

FEIGGDKKGKGTSLF

**>Pm011260 (PmSWEET4) locus=Pm3:9921623:9924001:+ [translate_table: standard]**

MVFSASHSVFIICRDAAGIAGNIFAFGLFLSPIHTYRRIIRNRSTEEFSG

LPYIYALLNCLICTWYGSPLVSSDNLLIMTVNSAGAVFQLVYIALFIIYA

EKSKKVRMLGFLLADFGLFAIIVFGSLQMTDLVMRQLIVGLLSCVSLISM

FASPMFIINLVIRTKSVEFMPFYLSLSTFLMSTSFFLYGIFNYDLFIYVP

NGIGTILGIVQLALYFYYKDSSKEDSREPLIAPYP

**>Pm013198 (PmSWEET5) locus=Pm4:2433448:2434735:- [translate_table: standard]**

MDTDMIRTIVGIIVHPDNLLVVTINGTGLVIEFIYIAIFFVFSPGKKRRN

IIIALLVEVVFFAVVVFITLHFFHDTKGRSMIIGILSIVFNIIMYNSPLT

VMKMVIKTKSVKYMPFYLSLANLCNGIVWTIYALLKFDPYLLLPNGLGAV

SGAVQIILYATYYKTTNWDEDG

**>Pm015728 (PmSWEET6) locus=Pm4:21122646:21124537:- [translate_table: standard]**

MVTADAARNVVGIIGTRPTFYRIIKKKTVEEFRPDPYIATVLNCLFWCLY

GMPFVHPDSLLVVTINGVGLVLELAYLAVFFIYAQSKGRKKVAFGLGFDV

VLFVAIAAISLTALHGTKKRSLLVGIVCDIFNIIMYGSPLTIMAKVIKTK

SVKYMPFYLSLTNFLNGCCWTAYALIKFDIYMLVSNGLGAVSGAIQLILY

AAYFKSTPKDDDDFAGKPSTEVQLSNTNAAAAASV

**>Pm017566 (PmSWEET7) locus=Pm5:12327097:12328384:+ [translate_table: standard]**

MAINFVVIFGVLGNVMSGLVYVSPANVFVRILRRRSTEEFESVPYVSKLL

NAYFWVYYGLIKPNSVLVATVNIFGAVVEIVFLTIFLLFAPPRMKARTAM

LVVALNVAFPAAAILLTQFLLHGDQRIDAAGLLCSIFSMIAYASPLSAMK

TVVALKSVEYMPFLLSFILFLNGGIWTLYSILAKDLFVGIPNGTGFLFGT

AQLILYAIYWKPSKPPKQVSDDLEDQQQHIREALIPASKPIIAE

**>Pm018875 (PmSWEET8) locus=Pm5:20984940:20990591:+ [translate_table: standard]**

MKSNEQLAFIFGLIGNIVSVMVFLAPIPTFYKIYKNKSSEGFQSTPYVVA

LLSAMLLLFYGVLKTNAALIISINVIGCVIEITYLIFYFVYASKKDKITT

MIQILVLNVAVFGLVVAVTFLLVGEDKRVSTVGWICAVFGVAVFAAPLLI

MRIVIQTKSVEYMPFYLSFSLTICATLWFLYGLFVKDYYIAHGGQHRLML

TQVLHSCCVTPSGFHPPSLHPSAPRSSLKSSSLFGEPLRQVPRSSLKVSK

TKQSSLVTRCAIGESLEVFLAKATPDKGLIRLLISMGEALRTISFKVRTA

ACGGTACINSFGDEQLAVDMLADKLLFEALSYSHFCKYACSEEVPELQDM

GGPVEGGFSVAFDPLDGSSIVDTNFTVGTIFGVWPGDKLTGITGRDQVAA

AMGIYGPRTTYVLAIKGFPGTHEFLLLDEGKWQHVKETTEIGEGKLFSPG

NLRATFDNPDYDKLINYYVKEKYTLRYTGGMVPDVNQILVKEKGVFTNVI

SPTTKAKLRLLFEVAPLGLLIENAGGYSSDGHQSVLDKVIVNLDDRTQVA

YGSKNEIIRFEEILYGSSRLKAGVPVGAAA

**>Pm019954 (PmSWEET9) locus=Pm6:436315:437664:- [translate_table: standard]**

MAAPDAFLLASVFGILGNIVAFMVYLAPLPTFYRIFKKKSTEGFQSIPYS

VALFSAMLMLYYAFLKTNAFMLITINSVGCIIETSFLVMYIIYAPAKTRI

YTAKLLVLFNAGVYGVIILSTYLIPNHFLRIKVVGWISVVFSVCVFAAPL

SIMRLVIRTRSVEFMSFPLSFCLTLCAVMWFFYGLLVRDLFIAAPNILGF

AFGLAQMIMYLMFKNSKKSILPEFSLNQIPNVVAVNDIVASDSQLKTKDT

KKSSEAEENQSTESMTNDSRAGDAV

**>Pm021931 (PmSWEET10) locus=Pm6:12459796:12461199:+ [translate_table: standard]**

MVNLEAIRIIVGIIGNVISFFLFTSPFTTFLKIIKQKSVGEFKADPYVAT

LLNCAMWSFYGMPFVHPDSVLVVTINGCGFVIELIYIAIFLTYSSNAKRR

WILIALLVEVIFFAVVVFVTLHFLHTTKGRSMIIGILCIVFNIIMYASPL

TIMKMVIKTKSVKYMPFTLSLANFCNGIVWLIYALIKFDPYILVPNGLGS

ISGLVQLILYATYYKSTNWDEDDEIQKSEVQLLDV

**>Pm022695 (PmSWEET11) locus=Pm6:19934418:19935334:+ [translate_table: standard]**

MLSFRMFGVKDFMNIFKFLFRLVFGNAIAFFLFLVPMTTLKRIMKKNKFT

EQYLSGIPYLMTLLLSAAWYDLPLVSPKNILVSTINTIGAAIEAIYLLIV

FLLAPKMEKDKILELLTFALSFKQSENLIQVDLFKIIK

**>Pm022696 (PmSWEET12) locus=Pm6:19944525:19945680:+ [translate_table: standard]**

MTLLNCLLSAWYGLPFVSPNNILVSTINGTGAAIEAIYVLIFIIFAPKRE

KFKILGLFTFVLALFSTVALVSVFALHSKARKLFCGLAATVFSIIMYGSP

LAIMSTVIKTKSVEFMPFFLSLFSFLCGTSWFIFGLLGHDPFVAVPNGFG

SGLGALQLVLYFIYRDLDSKGSTGSIKKPSSSSTAATADESMEMGFAEPH

QSKQSIAISGAQDGQP

**>Pm024167 (PmSWEET13) locus=Pm7:10796671:10798904:- [translate_table: standard]**

MVSADVVRTVVGVIGNVISLILFLSPVPTFVRIWKKGSVEQYSPAPYLAT

LANCMVWALYGMPMVHPHSILVVTINGSAIFIELSYIILFLIYASDKKQR

LKVLLILLVELVLMALLVLTVLILAHTYERRSLIVGIVCILCNIMMYASP

LAVMKLVITTKSVEFMPFCLSLFSFANGLAWFSFALIRFDIFLTIPNGLG

LLFGSGQLILYATYYKNTKRLLAERKARQVSLTEVVSDGDEPKKIGSTTQ

NGSAPHHGT

**>Pm024554 (PmSWEET14) locus=Pm7:13005181:13005663:- [translate_table: standard]**

MPFPLSFFLTLGAVTWFFYGLLIKDYNIAFPNILGFLFGIAQMVLYIVYK

NTKKVLEEQPKVQELSEHIIDVVKISSLVCPELSPVVLQPTLDXXXXGSE

YNRDG

**>Pm024555 (PmSWEET15) locus=Pm7:13012731:13014646:- [translate_table: standard]**

MTSSSAHSPLVIAFGILGNVVSFVVFLAPVPTFWRIYKKKSTEGFQSVPY

VFALFSAMIWIYYAFLKSDEFLLITINAFGCIIETIYISMYITYAPKQAR

VFALRLLLLVNFGGFCLILLLSHFLAQGPTRVEVLGWVCVAFSVSVFAAP

LSIMRVVIRTKSMPFSLSFFLTLSAVMWLFYGLLLKDLYVACPNILGFTF

GVAQMILYAIYRNKKTVLVEDQKLPVHKGDVVKQIQILSTTPEVEIQVQA

AAVSSHANTDNENCEQNKDQYVHPQTCNTEKIIGPSMPSQMVTCEV

**>Pm024712 (PmSWEET16) locus=Pm7:13852243:13854234:+ [translate_table: standard]**

MVDTGTIRTVVGIIADFKPDPYVATLLNCAMWVFYGMPFVHPDSILVITI

NGAGLVIEFIYITIFIIYSPGSKRKKIFIALVTEVIFFVIVVFVVLYCFH

TTKRRSLIVGIICIVFNILMYASPLTIMKMVIKTKSVKYMPFYLSLTNLL

NGVVWVVYALLKFDINILIPNGLGAISGIIQLILYATFYGTTRWDDDDDE

INSRSEVQMSNV

**>Pm030352 (PmSWEET17) locus=scaffold54:138478:139392:- [translate_table: standard]**

MLSFRMFGVKDFMNIFKFLFRVVFGGVFSVPVFFFSSTLDCSSDFVYRLM

PLYFDAGNGIAFFLFLVPMTTLKSIMKKNEFTEQYLSGIPYLMTLLLSAA

WYGLPLVSPKNILVSTINTIGAAIEAIYLLIVFLLAPKMEKDKILELLTY

TLSFKQSENPKKIKETLST
