# Supplementary material for: Genome-wide identification of the SWEET gene family mediating the cold stress response in Prunus mume
Source: PeerJ. 2022 May 3;10:e13273. doi: 10.7717/peerj.13273 (PMC9074862; doi:10.7717/peerj.13273)

**Domain architecture of *PmSWEETs***

The domain of *PmSWEETs* was predicted by SMART (http://smart.embl-heidelberg.de/).
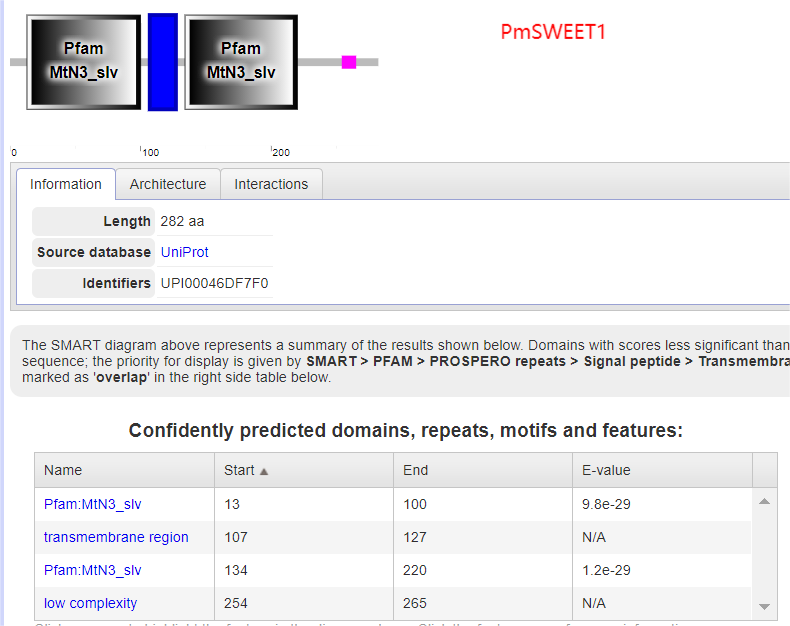


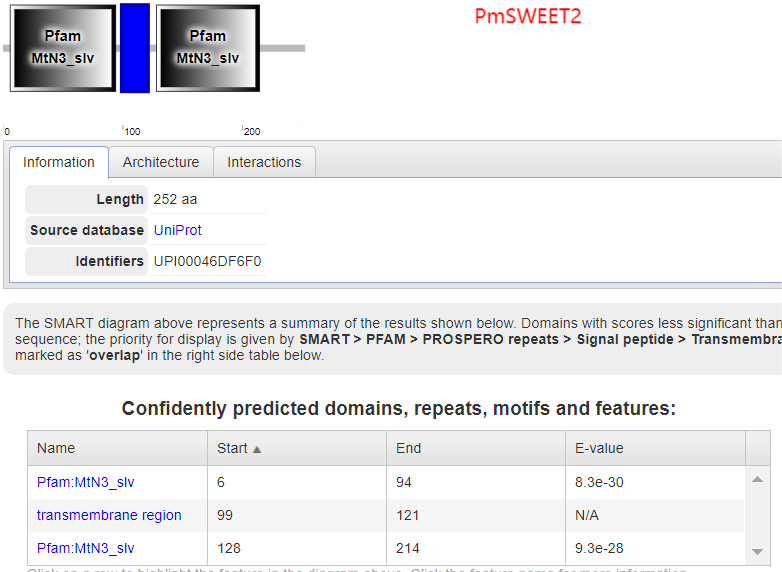


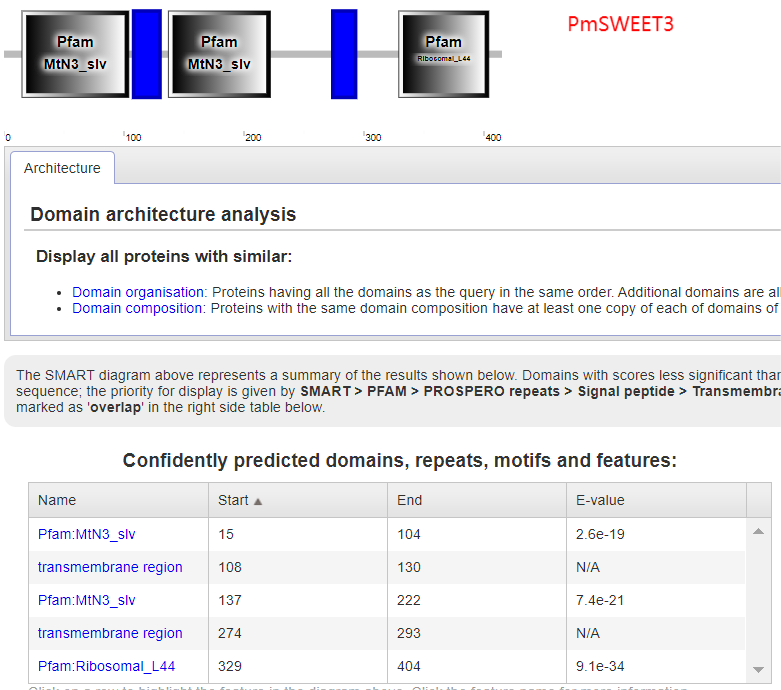

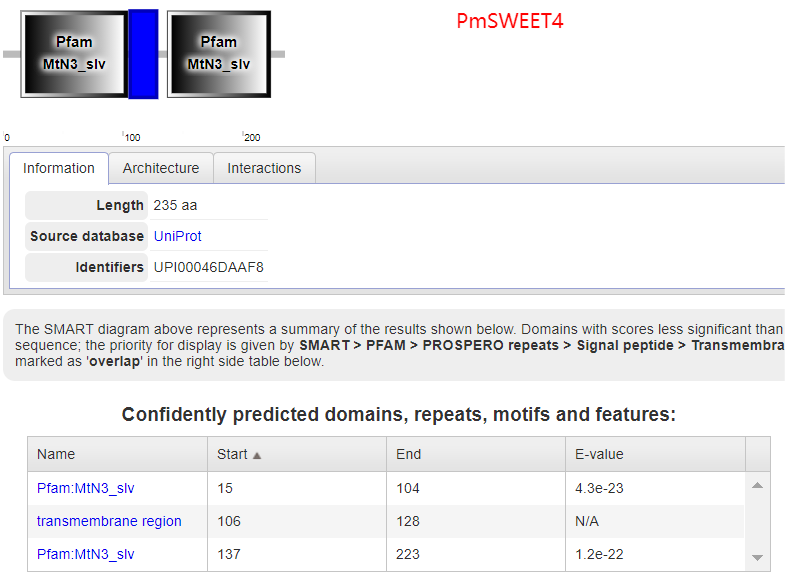

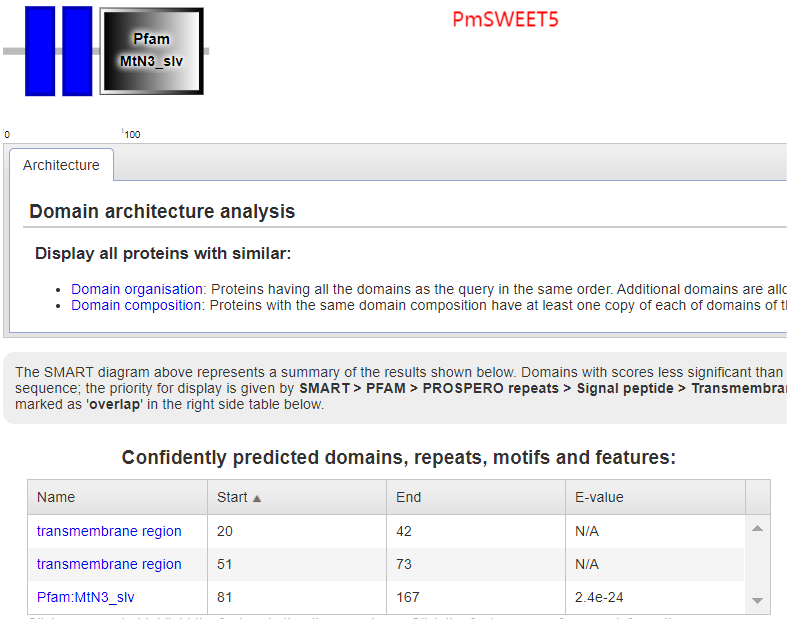

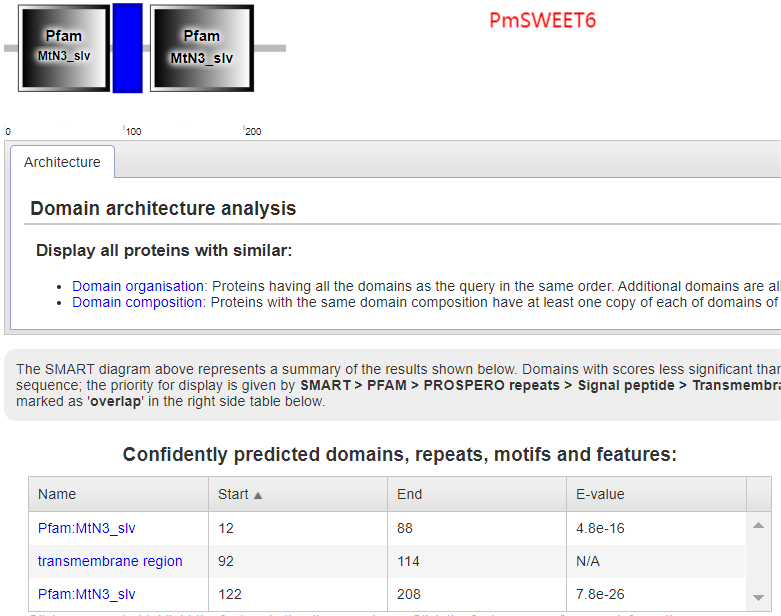

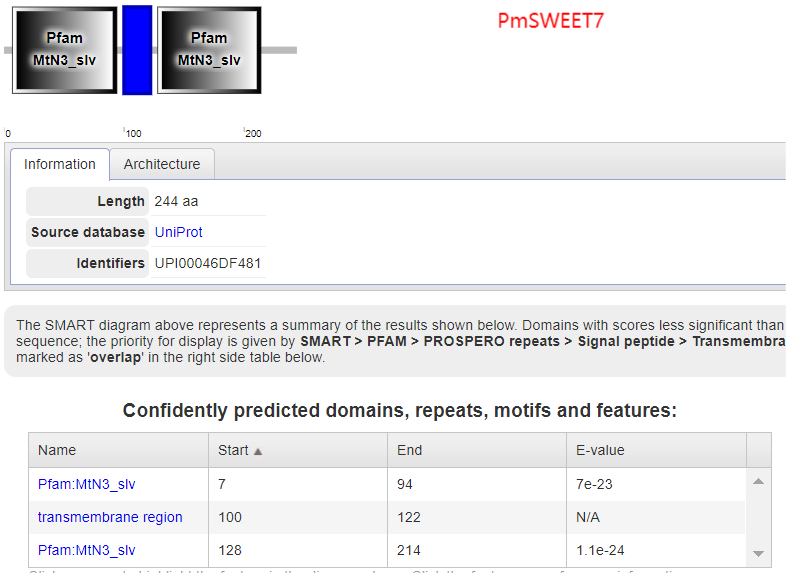


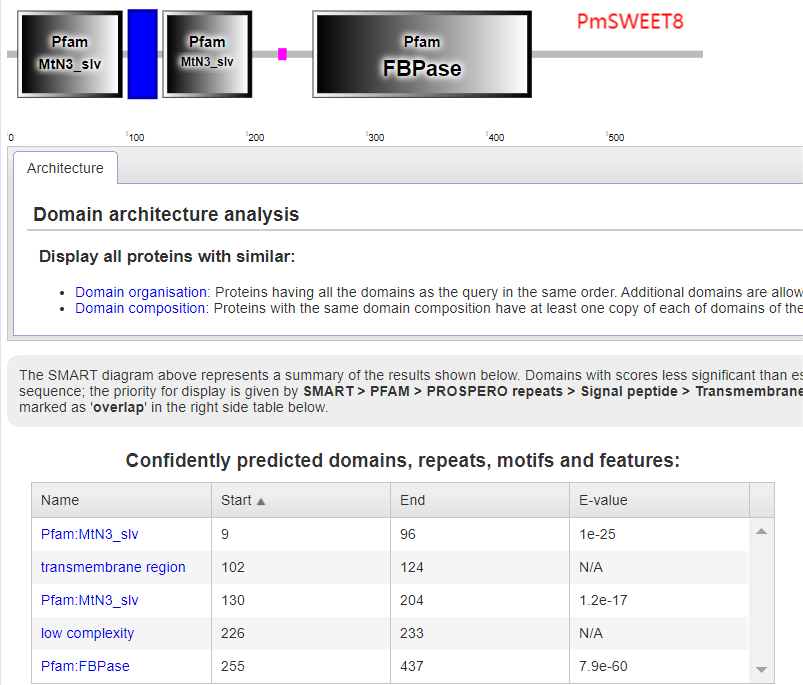


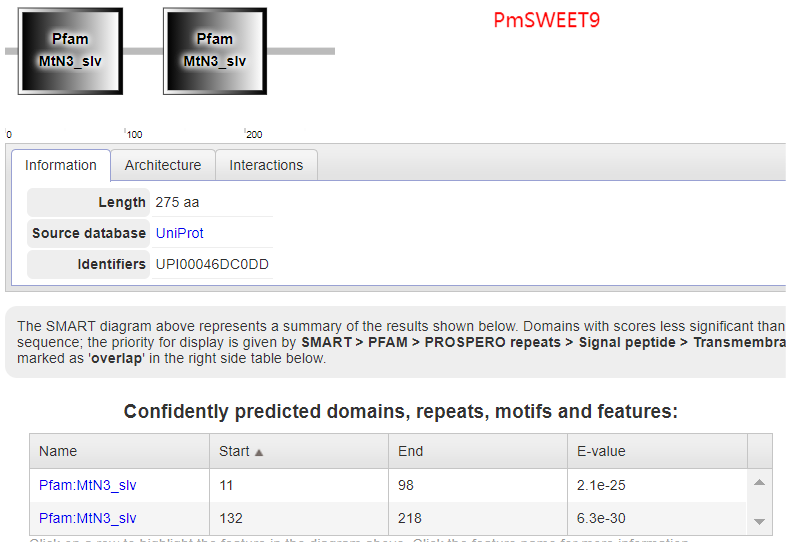

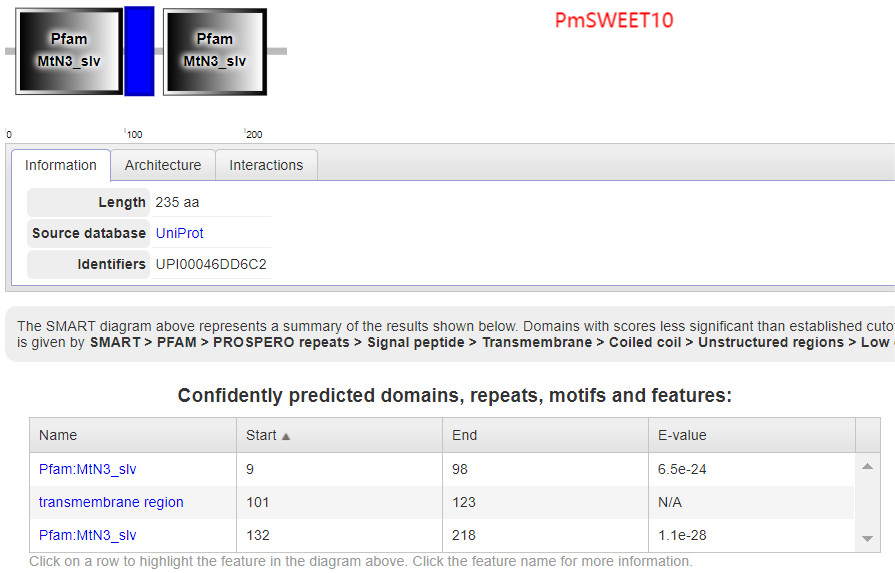

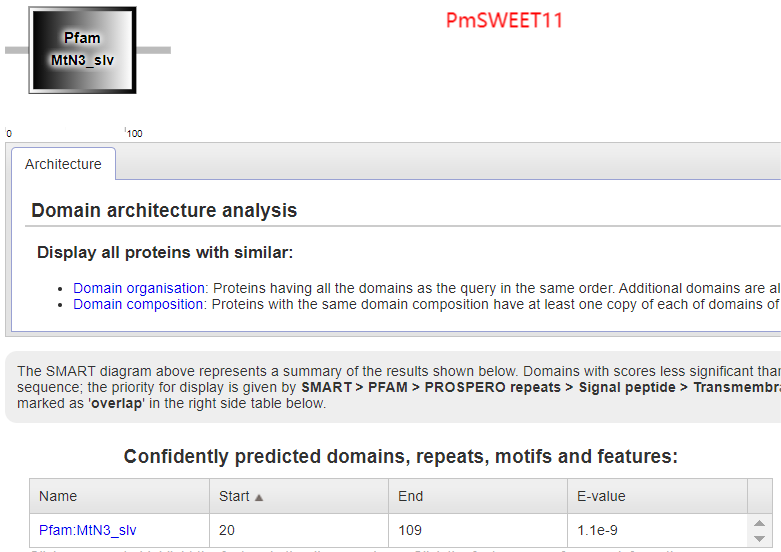

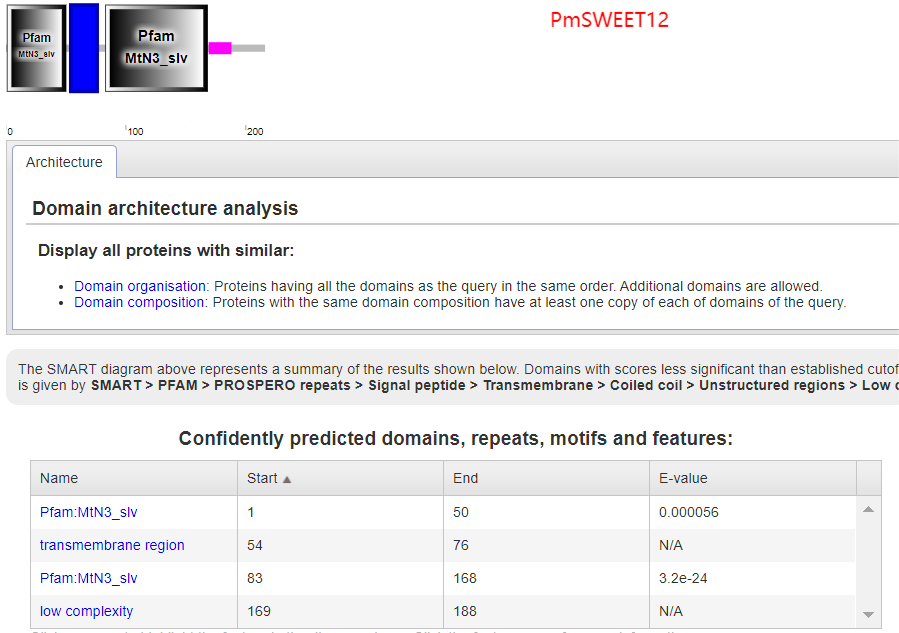

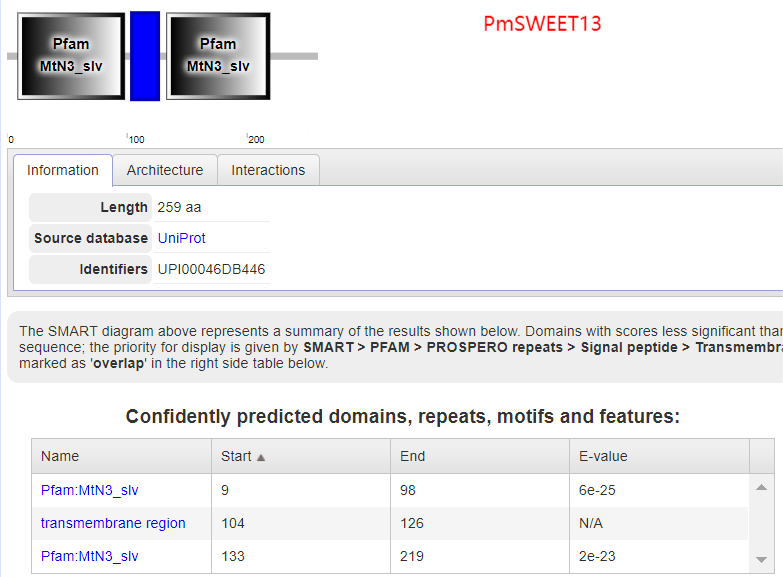

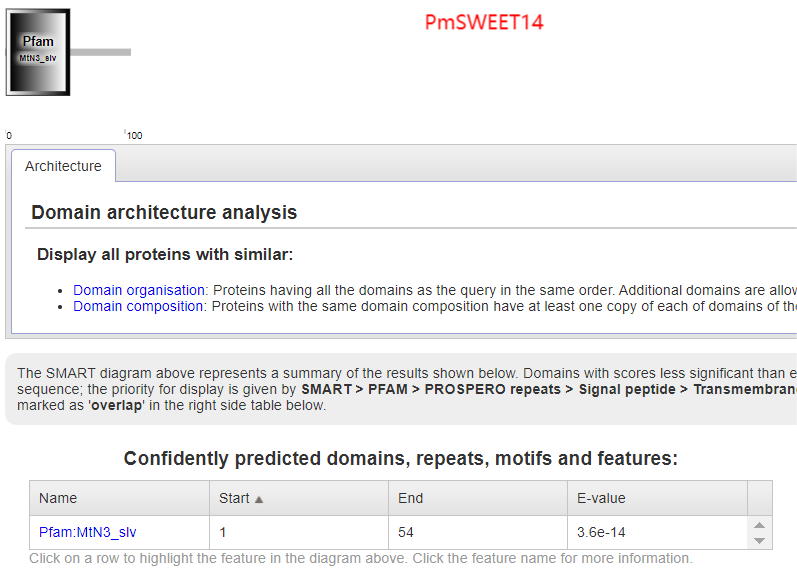


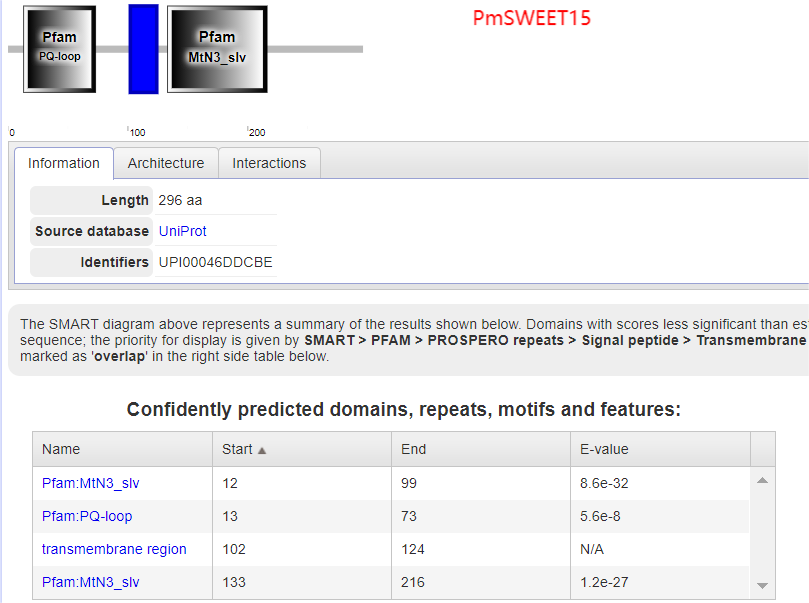

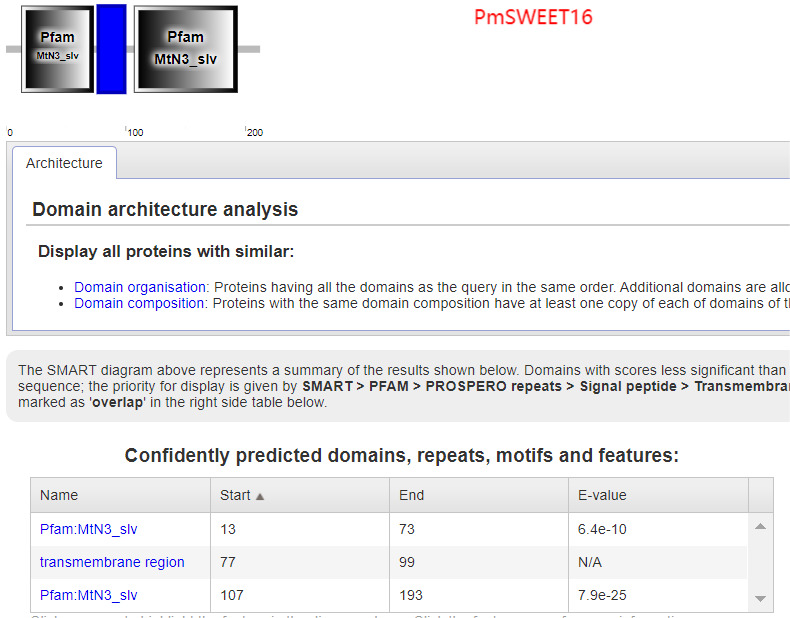

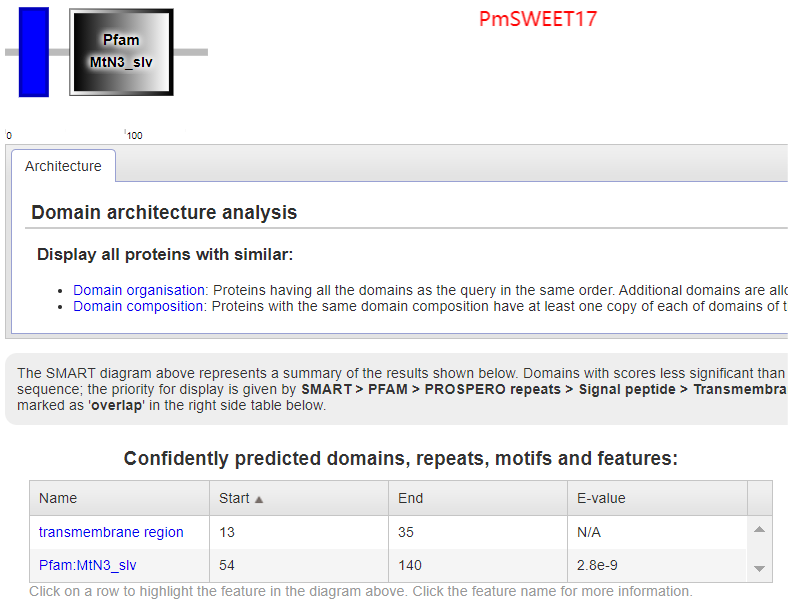

Supplement: Supplemental Information 6 [file peerj-10-13273-s006.docx]
